# mTORC1-Driven Protein Translation Correlates with Clinical Benefit of Capivasertib within a Genetically Preselected Cohort of *PIK3CA*-Altered Tumors

**DOI:** 10.1158/2767-9764.CRC-24-0113

**Published:** 2024-08-13

**Authors:** Constance A. Sobsey, Bjoern C. Froehlich, Georgia Mitsa, Sahar Ibrahim, Robert Popp, Rene P. Zahedi, Elza C. de Bruin, Christoph H. Borchers, Gerald Batist

**Affiliations:** 1 Segal Cancer Proteomics Centre, Lady Davis Institute, Jewish General Hospital, McGill University, Montreal, QC, Canada.; 2 Division of Experimental Medicine, McGill University, Montreal, QC, Canada.; 3 University of Victoria-Genome British Columbia Proteomics Centre, University of Victoria, Victoria, BC, Canada.; 4 Department of Biochemistry and Microbiology, University of Victoria, Victoria, BC, Canada.; 5 MRM Proteomics Inc., Montreal, QC, Canada.; 6 Department of Internal Medicine, University of Manitoba, Winnipeg, MB, Canada.; 7 Manitoba Centre for Proteomics and Systems Biology, Winnipeg, MB, Canada.; 8 Department of Biochemistry and Medical Genetics, University of Manitoba, Winnipeg, MB, Canada.; 9 CancerCare Manitoba Research Institute, Winnipeg, MB, Canada.; 10 Oncology R&D, AstraZeneca, Cambridge, United Kingdom.; 11 Gerald Bronfman Department of Oncology, Lady Davis Institute, Jewish General Hospital, McGill University, Montreal, QC, Canada.; 12 Department of Pathology, McGill University, Montreal, QC, Canada.; 13 Segal Cancer Centre, Jewish General Hospital, McGill University, Montreal, QC, Canada.; 14 McGill Centre for Translational Research in Cancer, Lady Davis Institute, Montreal, QC, Canada.

## Abstract

Capivasertib is a potent selective inhibitor of AKT. It was recently FDA approved in combination with fulvestrant to treat HR^+^, HER2-negative breast cancers with certain genetic alteration(s) activating the PI3K pathway. In phase I trials, heavily pretreated patients with tumors selected for activating PI3K pathway mutations treated with capivasertib monotherapy demonstrated objective response rates of <30%. We investigated the proteomic profile associated with capivasertib response in genetically preselected patients and cancer cell lines. We analyzed samples from 16 *PIK3CA*-mutated patient tumors collected prior to capivasertib monotherapy in the phase I trial. PI3K pathway proteins were precisely quantified with immuno-Matrix-Assisted Laser Desorption/Ionization-mass spectrometry (iMALDI-MS). Global proteomic profiles were also obtained. Patients were classified according to response to capivasertib monotherapy: “clinical benefit (CB)” (≥12 weeks without progression, *n* = 7) or “no clinical benefit (NCB)” (progression in <12 weeks, *n* = 9). Proteins that differed between the patient groups were subsequently quantified in *AKT1- or PIK3CA*-altered breast cancer cell lines with varying capivasertib sensitivity. The measured concentrations of AKT1 and AKT2 varied among the *PIK3CA*-mutated tumors but did not differ between the CB and NCB groups. However, analysis of the global proteome data showed that translational activity was higher in tumors of the NCB vs. CB group. When reproducibly quantified by validated LC-MRM-MS assays, the same proteins of interest similarly distinguished between capivasertib-sensitive versus -resistant cell lines. The results provide further evidence that increased mTORC1-driven translation functions as a mechanism of resistance to capivasertib monotherapy. Protein concentrations may offer additional insights for patient selection for capivasertib, even among genetically preselected patients.

**Significance:**

Capivasertib’s first-in-class FDA approval demonstrates its promise, yet there remains an opportunity to optimize its use. Our results provide new evidence that proteomics can stratify genetically preselected patients on clinical benefit. Characterization of the same profile in cell lines furnishes additional validation. Among *PIK3CA*-altered tumors, increased mTORC1-driven translation appears to confer intrinsic resistance. Assessing mTORC1 activation could therefore prove a useful complement to the existing genetic selection strategy for capivasertib.

## Introduction

The PI3K/AKT/mTOR signaling pathway has an established role in tumor cell proliferation, is mutated in more than 50% of breast cancers (1), and is the target of many new therapeutic agents (2). Although direct mutations of AKT are comparatively rare, overexpression and overactivation of AKT are key factors in cancer progression. In response to PI3K pathway activation, phospho-AKT promotes downstream oncogenic functions including cell growth, cell survival, and metabolic changes (3). Capivasertib (AZD5363, Truqap) competitively interacts with the ATP binding site of all three AKT isoforms (AKT1/2/3) and inhibits AKT’s catalytic activity with high potency and higher selectivity than previously developed compounds, resulting in the arrest of tumor cell growth (4).

Capivasertib in combination with fulvestrant was FDA approved in November 2023 for the treatment of hormone receptor (HR)-positive, HER2-negative advanced or metastatic breast cancer, with one or more confirmed genetic alterations associated with PI3K pathway activation (5)*.* FoundationOne was approved as a companion diagnostic assay for the associated genetic markers, which include mutations, copy-number variation, and other alterations, in *PIK3CA*, *AKT1*, or *PTEN* (5). The eligible cohort is currently limited to adults who progressed on endocrine-based therapy in the metastatic setting or who had recurrence during or within 12 months of completing adjuvant therapy. The approval of capivasertib plus fulvestrant therapy is based on results of the CAPItello-291 phase III trial, which demonstrated a dramatically reduced hazard ratio (HR) of 0.51 on the primary endpoint of progression-free survival (PFS) for combination therapy versus fulvestrant alone in patients with PI3K-altered tumors (5). However, an exploratory analysis of patients whose tumors did not harbor identifiable PI3K pathway alterations found a possible benefit for the addition of capivasertib in this cohort as well, as indicated by a HR of 0.61 to 1.02 [95% confidence interval (CI)] after excluding patients with unknown next-generation sequencing results.

Previous clinical trials have delivered varying results with respect to the power and necessity of genetic biomarkers for capivasertib. In phase I trials, heavily pretreated patients, patients selected for their tumors' activating *PIK3CA* and *AKT1* mutations had objective response rates (ORR) of 4% to 29% to capivasertib monotherapy (6–8). This represents a clinically meaningful response rate in a population of patients with advanced solid tumors who had previously progressed on standard therapies. However, higher clinical activity might be expected in a population carefully selected for a targeted agent. The FAKTION phase II trial (*n* = 140), which evaluated capivasertib plus fulvestrant in aromatase inhibitor-resistant estrogen receptor (ER)-positive breast cancer, initially found no effect of mutations on the response rate to combination therapy (9), whereas a subsequent updated analysis (*n* = 185) found that the benefit was greater in patients with activating mutations (10). In CAPItello-291, the clinical benefit (CB) rate for combination therapy of 56% among the subset of patients with PI3K-altered tumors (*n* = 289) was similar to the rate of 51% across the overall population (*n* = 708) enrolled in the study (5).

While the recent FDA approval doubtlessly weighed the results of all of these studies in the decision to guide patient selection with gene-based assays, we believe that this data—together with the fact that capivasertib is first-in-class—means that, like many new drugs, further investigation is merited into biomarkers to help optimize the effective use of capivasertib in clinical practice. Direct measurement of the proteome has shown unique potential for identifying and verifying active cancer-driving alterations that closely correspond to a tumors’ treatment sensitivity or resistance (11, 12). To evaluate the utility of proteomics for predicting treatment response to capivasertib, we utilized modern mass spectrometry–based methods to directly analyze the protein content of formalin-fixed, paraffin-embedded (FFPE) tumor tissues from patients enrolled in a phase I study of capivasertib’s antitumor activity. To further verify an association between the observed proteomic profile and capivasertib response, we applied corresponding targeted assays, validated in accordance with the NCI’s Clinical Proteomics Tumor Analysis Consortium (CPTAC) guidelines, to precisely quantify proteins of interest in analogous *PIK3CA*-altered breast cancer cell lines with varying sensitivities to capivasertib.

## Materials and Methods

Full descriptions of all methods are provided in the Supplemental Materials.

### Patient tumor samples

The study was conducted in accordance with the Declaration of Helsinki and approved by the Research Ethics Committee of the Jewish General Hospital (Project #2018-663 17-004, approved March 03, 2017) in Montreal, Quebec, Canada. Anonymized patient data and samples were obtained under site-specific Research Ethics Board approval as part of AstraZeneca’s registered international multicenter clinical trial of AZD5363 (NCT01226316, posted October 22, 2010; ref. 6). Written informed consent was obtained from all participants. De-identified samples and clinical data were provided for analysis under the conditions of a legal agreement between AstraZeneca, McGill University, University of Victoria, and the funders, in accordance with the patient consent form.

The sample set was drawn from patients with breast cancer whose tumor pathology was either ER positive (ER^+^) or HER2-positive (HER2^+^), or patients with gynecological (ovarian, cervical, or endometrial) cancers for whom no standard therapy was effective. For this cohort, eligibility was restricted to patients whose tumors contained known activating *PIK3CA* mutation(s) detected during screening. Tumor samples obtained at the time of screening were stored at ambient temperature as slide-mounted 4-µm-thick FFPE tumor tissue slices.

The enrolled patients received capivasertib 480 mg twice daily for 4 days followed by 3 days off, repeated in 21-day cycles. Tumor volume was measured according to RECIST v1.1 criteria within 28 days of the start of treatment and at specified timepoints after the start of treatment (i.e., weeks 6, 12, 18, 24, etc.). Target lesion response was categorized into complete response (CR), partial response (PR, ≥30% decrease), stable disease (SD, volume ± <30%), or progressive disease (PD, ≥30% increase; ref. 6). Treatment was continued as tolerated, until evidence of disease progression. Standardized, anonymized response data were used to classify patient drug response based on PFS. CB was defined as PFS observed for a minimum of 12 weeks after starting capivasertib. “No CB” (NCB) was defined as less than 12 weeks of observed PFS while receiving capivasertib.

### Cell line samples

Well-characterized hormone-receptor positive, *AKT1*- or *PIK3CA*-altered breast cancer cell lines were selected for analysis. HCC-1428 (RRID:CVCL_1252) was purchased from ATCC (CRL-2327, via Cedarlane, Feb 2022). EFM-19 (RRID:CVCL_0253) was purchased DSMZ (ACC 231, July 2022). ZR-75-30 (RRID:CVCL_1661) and MCF-7 (RRID:CVCL_0031) were gifts from the laboratory of Dr. Mark Basik at the Segal Cancer Centre (received May 2022). Cell lines underwent short tandem repeat DNA profiling and mycoplasma detection/elimination prior to the beginning of the experiments, which were conducted June to November 2022. All cell lines were cultured in RPMI-1640 with 10% or 15% FBS, according to manufacturer directions, with 1% P/S, for fewer than 8 passages. Samples of each cell line were collected prior to capivasertib exposure, washed with PBS, and stored at −80°C until analysis. Each cell line was tested for sensitivity to capivasertib using a standard alamarBlue cytotoxicity assay, based on at least three independent replicates.

### Protein extraction, digestion, and immuno-Matrix-Assisted Laser Desorption/Ionization mass spectrometry (iMALDI-MS)

Tumor samples were processed and analyzed using a multistep workflow (Supplementary Methods S1), beginning with xylene deparaffinization and ethanol rehydration. Samples were then extracted by high-temperature incubation and sonication. The concentration of total protein in each sample was quantified by Pierce bicinchoninic acid protein assay kit with a ThermoFisher MultiSkan Go spectrophotometer.

Quantitation of AKT by immuno-matrix-assisted laser desorption/ionization (iMALDI)-MS was performed as previously described (13). Diluted sample aliquots containing 10 µg of total protein were prepared in an automated fashion on an Agilent Bravo Liquid Handling Robot. The calibration curve was prepared by spiking known quantities (0–20 fmol) of unlabeled standard peptide into 10 µg of bovine serum albumin. Each tumor or calibration sample was denatured with sodium deoxycholate (DOC), reduced with tris(2-carboxyethyl)phosphine (TCEP), alkylated with iodoacetamide (IAA), and quenched with dithiothreitol (DTT). Digestion was performed with tosyl phenylalanyl chloromethyl ketone (TCPK)-treated trypsin (substrate-to-enzyme ratio of 2:1, 1 hour, 37°C), prior to quenching with tosyllysine chloromethylketone (TLCK). A phosphatase-based phosphopeptide quantitation (PPQ) method, as described in Domanski and colleagues (14), was performed by incubating aliquots with or without alkaline phosphatase (1U/µg total protein, 2 hours, 37°C). Stable isotope-labeled internal standard peptides were added to each digested sample.

Immunoenrichment was achieved by tumbling samples overnight at 4°C with antipeptide antibodies (Signatope GmbH) coupled to magnetic beads. The AKT-depleted supernatant from this step was retained; a portion was reserved for global proteome analysis by nano-LC-Orbitrap MS. The remainder was sequentially enriched using antipeptide antibodies for PTEN and PI3K p110α to enable quantitation via our previously validated iMALDI method (15). Beads were washed prior to spotting on a MALDI target. α-Cyano-4-hydroxycinnamic acid (HCCA) MALDI matrix was applied to the dried spots, which were stored at room temperature until analysis.

Mass spectra were acquired on a Bruker Microflex LRF benchtop MALDI-TOF-MS (RRID:SCR_018696). Data analysis was performed in FlexAnalysis 3.4 with Savitsky-Golay smoothing, baseline subtraction, and automated peak picking. Calibration curves were generated using a linear regression with a 1/x^2^ weighting. Assay performance was confirmed with quality control samples (QC samples; Supplementary Validation Data S1).

### Nano-LC-Orbitrap MS analysis, protein identification and quantification

A 2-µL aliquot of the AKT-depleted supernatant from each sample’s immunoenrichment, corresponding to 117.5 ng of total protein digest, was reserved for label-free quantitation of the global proteome. Samples were prepared using custom StageTips for desalting before drying under vacuum and resuspension in 0.1% formic acid for injection.

Online liquid chromatography was performed on an Easy-nLC 1200 equipped with a precolumn (particle size, 3 µm; 2 cm × 75 µm inner diameter) and a nanoscale main analytical column (particle size, 2 µm; 25 cm × 75 µm inner diameter), both AcclaimPepMap100 C18 (ThermoFisher Scientific). Elution was performed over a 50-minute gradient (flow rate, 300 nL/minutes; 20°C; mobile phase A: 0.1% formic acid; mobile phase B: 84% acetonitrile, 0.1% formic acid).

Mass spectra were acquired on a Q-Exactive Plus MS with a Nanospray Flex ion source (RRID:SCR_020556, ThermoFisher Scientific). Using a data-dependent acquisition (DDA) method, the 15 most abundant precursor ions (charge states: 2+ to 4+) were selected for MS/MS fragmentation (dynamic exclusion, 40 seconds). Full scan MS were acquired for the mass range from m/z 350 to m/z 1,500 (resolution, 70,000; AGC target, 1 × 10^6^; max injection time, 50 ms). MS2 spectra were acquired following HCD fragmentation (normalized CE, 28), with an isolation width of 1.2 Da (resolution, 17,500; AGC target, 2 × 10^4^; max injection time, 64 ms).

MS raw global proteome data were processed using Proteome Discoverer 2.4 (RRID:SCR_014477, Thermo Scientific). Database searches were performed using SequestHT (RRID:SCR_000286) and a human Swiss-Prot database (RRID:SCR_021164, January 2019; 20,414 target entries), assuming a maximum of 1 missed tryptic cleavage. Mass tolerances were 10 ppm for precursor ions and 0.02 Da for product ions. Scaled, normalized, abundances were exported to Microsoft Excel (RRID:SCR_016137), and filtered for protein IDs with ≥2 unique peptides.

### LC-MRM-MS assays

Targeted multiplexed UPLC-MRM-MS assays were developed and optimized for 54 proteins of interest using synthetic proteotypic peptides for calibration and corresponding stable isotope-labeled (SIS) peptides as internal standards for quantitation (Sobsey and colleagues, submitted). Assays were characterized in accordance with the CPTAC guidelines (16).

Cell line samples underwent extraction and total protein quantitation using methods similar to those used on the deparaffinized patient tumor samples. Aliquots of 80 µg of total protein were manually prepared for in-solution digestion by denaturation with DOC, reduction with TCEP, alkylation with IAA, and quenching with DTT. Tryptic digestion was performed at a substrate-to-enzyme ratio of 20:1 (Worthington, TCPK-treated, 17 hours, 37°C), prior to quenching on ice, and spiking with SIS peptides. Solid Phase Extraction (Oasis SPE HLB 1 cc cartridges, 10 mg sorbent) was performed according to manufacturer directions on a vacuum manifold. Eluates were dried under vacuum and reconstituted in H_2_O, 0.1% formic acid for injection. The calibration curve was prepared by adding known equimolar quantities (0–100 fmol on-column) of 54 unlabeled synthetic peptides into digested BSA spiked with SIS peptide mix. A previously quantified pooled cell lysate sample was repeatedly analyzed as a quality control.

Samples and calibration standards were analyzed via 10 μL injections (20 µg total protein digest on-column) on an Agilent 1290 Infinity liquid chromatography system (RRID:SCR_019375) with a Zorbax Eclipse plus C18 column (RRHD; particle size, 1.8 µm; internal diameter, 2.1 × 15 mm; 50°C; flow rate, 0.35 mL/minutes) coupled to a 6495B triple quadrupole mass spectrometer (Agilent Technologies, Santa Clara, CA). Elution was performed over a 46-minute gradient (B: 2%–80%; mobile phase A: H_2_O, 0.1% formic acid, mobile phase B: acetonitrile, 0.1% formic acid). Mass spectra were acquired in positive ion mode using a scheduled MRM-MS method targeting ≥3 transition pairs per peptide. Data were processed in Skyline (RRID:SCR_014080) for peak integration and quantitation.

### Data analysis and bioinformatics

Descriptive statistics and protein concentration data were analyzed in Microsoft Excel (RRID:SCR_016137). Group differences were assessed using the common *t* test and nonparametric tests to address the possibility of non-normal distributions in the protein concentration data. Multivariate statistical analysis was performed using MetaboAnalyst (RRID:SCR_015539; ref. 17). Volcano plots, Principal Component Analysis, Partial Least Squares Discriminant Analysis, Variable Importance in the Projection (VIP) scores, heatmaps, and hierarchical clustering were generated based on normalized data. StringDB was used for network analysis, to identify clusters of related proteins, and to identify related publications (18). Cytoscape (RRID:SCR_003032) was used to visualize the regulation of proteins within the network (19). QIAGEN Ingenuity Pathway Analysis (IPA; RRID:SCR_008653) software was used to map the label-free quantification data to canonical pathways. QC analyses confirmed the integrity of the data following batch integration, normalization, and scaling of features (Supplementary Validation Data S2).

### Data availability

Individual participant data associated with the clinical trials sponsored by AstraZeneca can be requested by qualified researchers via the request portal, with request evaluated according to the AstraZeneca disclosure commitment (https://astrazenecagroup-dt.pharmacm.com/DT/Home) and Responsible Data Sharing Principles. Global proteomics spectra and associated were deposited to the ProteomeXchange Consortium (https://www.proteomexchange.org/) via the PRIDE partner repository with the dataset identifier PXD052949. Samples of the polyclonal antibodies for AKT1 and AKT2 can be requested through the Segal Cancer Proteomics Centre (Montreal, QC, Canada). LC-MRM-MS assay information and validation data are publicly available in the CPTAC Assay Portal (https://assays.cancer.gov/available_assays, IDs: 6077-6129; see Supplementary Methods S1 for links to each assay). MRM-MS assay kits including the required peptide standards and instrument files are now commercially available from MRM Proteomics Inc. (Montreal, QC, Canada).

## Results

Slide-mounted FFPE tumor samples were obtained from patients with activating *PIK3CA* mutations in their ER^+^ or HER^+^ breast or gynecological cancers, respectively, prior to treatment in a multicenter phase I clinical trial of capivasertib (NCT01226316). Twenty-three 4-μm slides, representing 16 patient tumors, yielded sufficient material (≥25 μg total protein/slide) for the analyses. Patients were coded as “CB” (≥12 weeks of PFS after treatment initiation; *n* = 7) or “NCB” (<12 weeks PFS; *n* = 9) as described in the “Methods” section. As shown in Table 1, the two groups had similar overall clinical characteristics, but the CB group had a greater proportion of samples derived from metastatic sites compared to the NCB group.

**Table 1:** Descriptive statistics for the patient samples analyzed

	Clinical Benefit (CB)	No Clinical Benefit (NCB)
	Group (*n* = 7)	Group (*n* = 9)
Clinical Information		
Breast (*n* =__)	3	4
Gynecological (*n* =__)	4	5
Patient weight (kg)	69.9 ± 18.8	58.9 ± 10.2
Patient age (years)	63.0 ± 10.9	56.7 ± 9.4
WHO Performance Status		
PS 0 (*n* =__)	3	5
PS 1 (*n* =__)	4	4
Metastatic sites at enrollment	2.7 ± 1.0	3.4 ± 1.6
Previous lines of treatment		
≤2	1	3
≥3	4	5
Not specified	2	1
Capivasertib Response		
Adjusted progression-free survival (weeks)*	median: 21.1	5.6
survival (weeks)*	(95% CI: 13.0 to 32.3)	(95% CI: 5.3 to 6.1)
Best change in tumour volume from	median: −11.9	median: 28.6
baseline (RECIST %)**	(95% CI: −52.2 to −8.1)	(95% CI: 2.3 to 35.9)
Sample Characteristics		
Sample site*	4	4
Metastatic (*n* =__)	5	1
Primary (*n* =__)	2	8
Storage time (years)	7.3 ± 0.7	7.6 ± 1.4
Tissue area (mm2)	330.7 ± 116.8	339.5 ± 151.3
Cellularity		
Tumour cells (%)	62.9 ± 11.6	65.9 ± 22.4
Necrosis (%)	3.9 ± 3.2	5.0 ± 2.7

Values are given as mean ± standard deviation unless otherwise specified. All patients were female.

*Denotes statistical significance at *P* < 0.05 (*t* test unequal variance, two-tailed), **P < 0.01.

### Tumor AKT protein concentrations do not differ between CB and NCB groups

Twenty-two slides representing 16 patient tumors were analyzed by iMALDI-MS. The lower limits of quantitation (LLOQ) for AKT1 and AKT2 were 0.3 and 0.6 fmol on-spot, respectively. Among the *PIK3CA* mutation-positive tumor samples with AKT data (CB *n* = 7, NCB *n* = 5), the amount of AKT present per 10 µg total protein ranged from 0.6 to 4.5 fmol for AKT1 and 0.6 to 2.0 fmol for AKT2. For tumors from which multiple slides were analyzed, the AKT concentrations showed good agreement across biological replicates, even among non-adjacent slices (*R*^2^ > 0.95). Most samples did not show sufficient phosphorylation (>30%) of AKT1-Ser473 or AKT2-Ser474 to be quantified by the PPQ assay, with measurable phosphorylation observed in only three tumors (Supplementary Fig. S1). Using the AKT-depleted digest from the AKT iMALDI assay, PTEN and PI3K p110α were quantified in 14 patient tumors (CB *n* = 7, NCB *n* = 7). Within the patient sample set, the PTEN concentration values quantified by iMALDI correlated well with the PTEN histochemical scoring assessment (H-score) previously determined by immunohistochemistry (*R*^2^ = 0.86; Supplementary Fig. S2).

**Figure 1:**
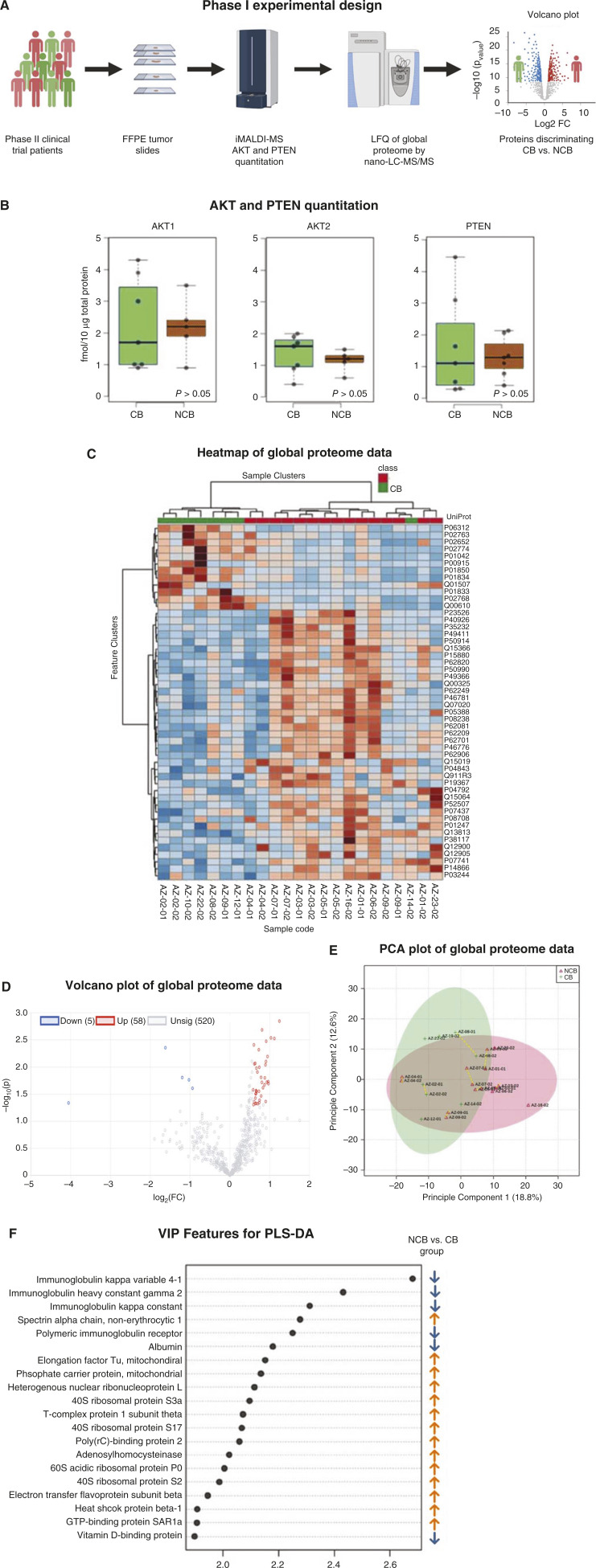
Statistical analysis of targeted and global proteome comparing protein expression in analyzed FFPE tumor tissues from the CB (green) and NCB (red) groups. **A,** Schematic of experimental design for phase I of the study. **B,** Results of targeted quantitation by iMALDI-MS. Boxplots of protein concentrations in the CB (green) vs. NCB (red) groups. Each point represents one patient tumor, averaged for multiple slides. *P*-values are given for a two-tailed *t* test. **C,** Heatmap of 50 selected proteins with highest differential expression between groups showing their normalized LFQ abundances across both analyzed batches. *n* = 23. Arbitrary identifiers are included for the purpose of identifying replicates from the same tumor. **D,** Volcano plot showing the fold change (log_2_FC) of all protein expression features vs. *P*-value (−log_10_*P*). **E,** PCA. Proximity of sample replicates originating from the same patients is indicated with a (dashed yellow line). **F,** Features ranked by VIP based on their contribution to the discrimination between the CB and NCB groups in the PLS-DA. The arrows on the right of the VIP Feature list show relative expression of that protein in the NCB group vs. the CB group.

Using our quantitative iMALDI-MS assay, we observed significant inter-tumor variability in the amount of AKT1 and AKT2 protein, with as much as a 4-fold difference among PIK3CA-mutated tumors. However, total AKT1, AKT2, and PTEN protein concentrations did not differ significantly between the CB and NCB groups (Wilcoxon rank test, *P* > 0.05; Fig. 1A). We separately quantified pAKT1-Ser473 and pAKT2-Ser474 proteins using a mass spectrometry assay with high isoform specificity. However, only three samples showed pAKT stoichiometry sufficient to be precisely quantified by the assay.

We also quantified PTEN protein since it is the dominant regulator suppressing AKT phosphorylation in *PIK3CA*-mutated cells. All three samples with >30% pAKT1 or pAKT2 had low PTEN values (<0.36 fmol PTEN/10 μg total protein, in Q1 of the measured values), as did one other sample with 22% pAKT1, which appeared elevated but fell below the assay’s LLOQ. Nonetheless, PTEN expression levels did not differ significantly between the treatment response groups. For PI3K p110α, the large number of samples with concentrations below the lower limit of quantitation of the assay prevented a meaningful comparison between the groups. AKT2 was slightly higher in the metastatic tumors as compared to primary tumors, but the difference did not reach statistical significance (*p* = 0.09; Supplementary Fig. S3).

### Proteins associated with oncogenic functions of AKT are differentially expressed between the CB versus NCB groups

Using a new serialized workflow, we acquired global proteomic data using nano-LC-MS-MS for 23 distinct tumor slides from 15 patient tumors (CB *n* = 6, NCB *n* = 9). The workflow permitted further analysis of previously analyzed samples from small aliquots of AKT-depleted supernatant, each containing approximately 107 ng of digested protein. We were able to achieve label-free quantification (LFQ) of up to 1,455 proteins at a false discovery rate of <1%, with a minimum of two unique peptides per protein. A total of 578 proteins were quantified across all samples for further statistical analysis. In light of the higher proportion of samples from metastatic sites in the CB group, the proteomic profiles of metastatic versus primary tumors were compared (Supplementary Fig. S3). The differences in metastatic versus primary tumors identified by PLS-DA were attributable to known markers of metastasis, indicating that biological relationships were preserved following batch integration and data normalization.

As shown in Fig. 1, unsupervised statistical analysis of the global proteome data by hierarchical clustering and by principal component analysis (PCA) confirmed the high similarity of replicates from the same tumor. Supervised statistical analysis with Partial Least Squares–Discriminant Analysis (PLS-DA) resulted in a clear separation of the CB and NCB groups, with little overlap. A total of 53 proteins had abundances that differed between the CB and the NCB group with fold-changes of ≥1.5 and Wilcoxon rank test *P*-values of ≤0.05 (Supplementary Table S1).

Of these 53 proteins, the five proteins that were upregulated in the CB group included four immunoglobulins and serum albumin. The five proteins whose expression was higher in the CB group were serum albumin (ALB), polymeric immunoglobulin receptor (PIGR), and some immunoglobulin subunits (IGHG2, IGKC, IGKV4-1). This is aligned with previous evidence linking systemic hypoalbuminuria with reduced PFS and overall survival in patients treated with TKIs (20, 21). High levels of serum albumin are also associated with reduced TNFα and pro-inflammatory cytokine signaling, diminished gluconeogenesis, and increased activation of AKT to promote cell survival (22). This is consistent with the observation that the CB group had reduced levels of CPNE-1 (suggesting lower TNFα signaling) and inflammatory proteins (ILF-2, ILF-3). There is mixed evidence about the role of PIGR in cancer, but some evidence suggests that PIGR promotes oncogenic AKT activity with downstream effects on GSK3B/β-catenin that are reversed by AKT inhibition (23).

The 48 proteins that were downregulated in the CB group versus the NCB group cluster to three main nodes. The largest group includes 13 structural subunits of ribosomes. A related, strongly interconnected, node relates to regulation and processing of mRNA and DNA. The final node consists of 11 differentially expressed mitochondrial proteins. Taken as a whole, the nodes suggest strongly increased translational activity in the NCB group together with the associated energetic demands. GTP-binding protein SAR1a was the only one of the 53 differentially expressed proteins that overlapped with the proteins differentiating metastatic from primary tumors.

**Figure 2:**
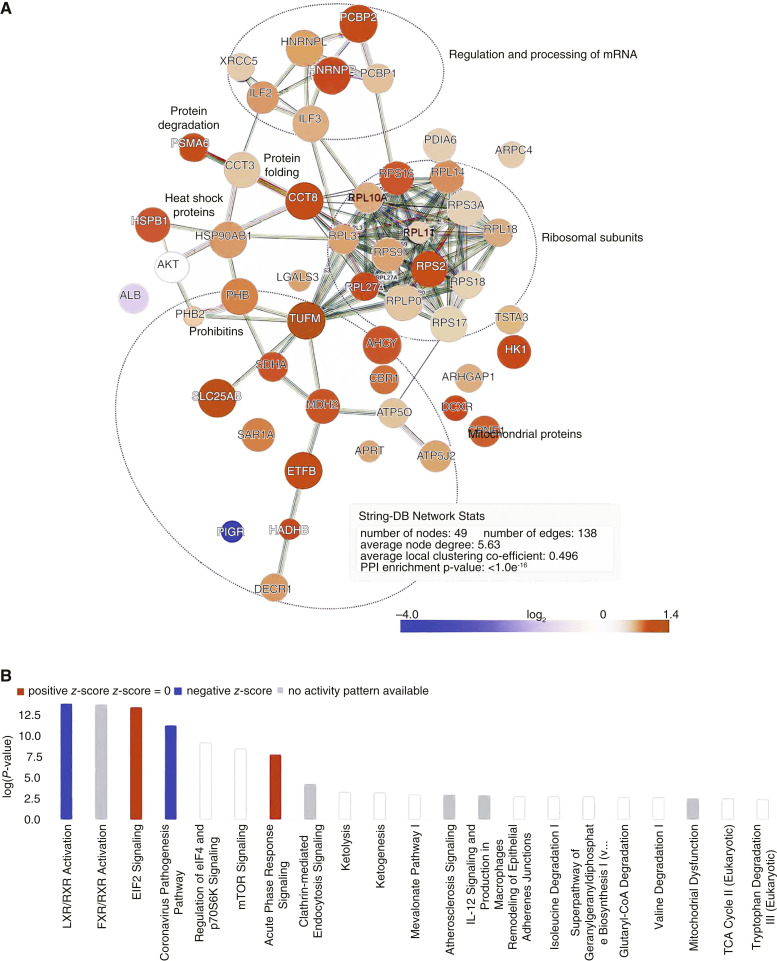
Mapping of global proteomics results from CB vs. NCB groups to protein networks and pathways. **A,** Network analysis via String-DB plot of high-confidence protein–protein interactions among proteins that are significantly different between CB and NCB group. Edges represent lines of evidence of protein–protein interactions. Line colors indicate different evidence types: known interactions from curated databases (teal) or published experiments (pink), predicted interactions based on gene location (green), fusion (red), or co-occurrence (blue), or observed co-expression (black), protein homology (purple), or textmining relationships (lime). Major clusters are labeled according to shared features within the cluster. Nodes are overlaid from the Cytoscape visualization, with fold-change shown by color. Node size increases as the *P*-value decreases. **B,** Top 20 canonical pathways that are significantly differentially activated between clinical benefit (CB) and no clinical benefit (NCB) groups, based on assessment with Fisher’s exact test in Qiagen IPA. The height of the bar corresponds to the confidence of an association, with a threshold of *P* < 0.01. IPA’s *Z*-score indicates the direction of regulation and extreme *z*-scores are depicted with increased color intensity. Orange bars represent increased activation in the NCB group whereas blue bars represent upregulated activity in the CB group. White bars indicate pathways with fewer than four mapped proteins or *z*-scores close to zero, indicating that the direction of regulation of individual pathway members does not strongly match a prespecified pattern. Gray bars indicate pathways for which no prediction can be made due to available evidence in the database.

Protein network analysis of the 48 downregulated proteins plus AKT1, using the String-DB, found 138 high-confidence interactions between proteins within this group (Fig. 2A). The observed protein–protein interaction enrichment *P*-value of <1.0e^−16^ provides strong evidence for a meaningful biological relationship between the proteins of interest in this dataset. Network analysis shows an enrichment of ribosomal proteins, mitochondrial proteins, and proteins involved in mRNA processing.

### Proteins differentially expressed between the CB versus NCB groups map to translational control pathways

To better understand the pathways responsible for the group differences observed, QIAGEN’s IPA software was used to systematically map the differentially expressed proteins, fold-changes, and *P*-values to canonical pathways and to statistically assess the observed patterns of regulation. Pathway enrichment analysis (Fig. 2B) identified a systematic enrichment of proteins in pathways associated with translational activity (e.g., EIF2 signaling, eIF4/p70S6K, mTOR pathway), inflammation (acute phase response signaling, LXR/RXR activation), cancer cell motility/invasion (actin cytoskeleton signaling), and altered glucose metabolism (gluconeogenesis, glycolysis). LXR/RXR was activated in the CB group. LXRs regulate glycolysis, lipid hemostasis, and possibly immune functions by enhancing the activity of GSK3β, which activates AKT and phosphorylates pathway proteins upstream (*RICTOR*, *PTEN*) and downstream (*TSC*) from AKT (24). Proteins belonging to translational control pathways directly downstream from AKT—–eIF4/p70S6K and mTOR signaling—were enriched among the proteins of interest. Expression is upregulated for the associated proteins in the NCB group, though IPA could not statistically confirm the direction of regulation.

The EIF2 signaling pathway is of particular interest in this context. eIF2α (EIF2S1) is a master regulator of translation dysregulation in cancer. By modifying the efficiency of translation initiation, PERK-phosphorylated eIF2α can selectively decrease or enhance translation, resulting in the uncoupling of mRNA abundance from protein expression for up to 90% of transcripts (25). Since eIF2α works hand-in-hand with AKT to control cell fate decisions, sending compensatory prosurvival signals under conditions of cell stress, eIF2α signaling has been previously identified as a possible mechanism of resistance to AKT inhibitors (25–27). Furthermore, the elevated expression of ribosomal and translational proteins observed in the NCB group could help sustain eIF2α phosphorylation by limiting ER stress that could trigger the unfolded protein response and sensitize the cells to AKT inhibition (28).

However, closer examination of the data shows that IPA defined its EIF2 pathway to include ribosomal proteins (RPL10A, RPL14, RPL18, RPLP0) that their EIF4 pathway does not. This led to statistical prioritization of EIF2 signaling in IPA but the authors could not find published evidence of a specific association of EIF2 signaling with these RPLs; in fact, the relationship appears to be mediated by EIF4.

### Targeted analysis in breast cancer cell lines reproduces the proteomic profile seen in patient samples

**Figure 3:**
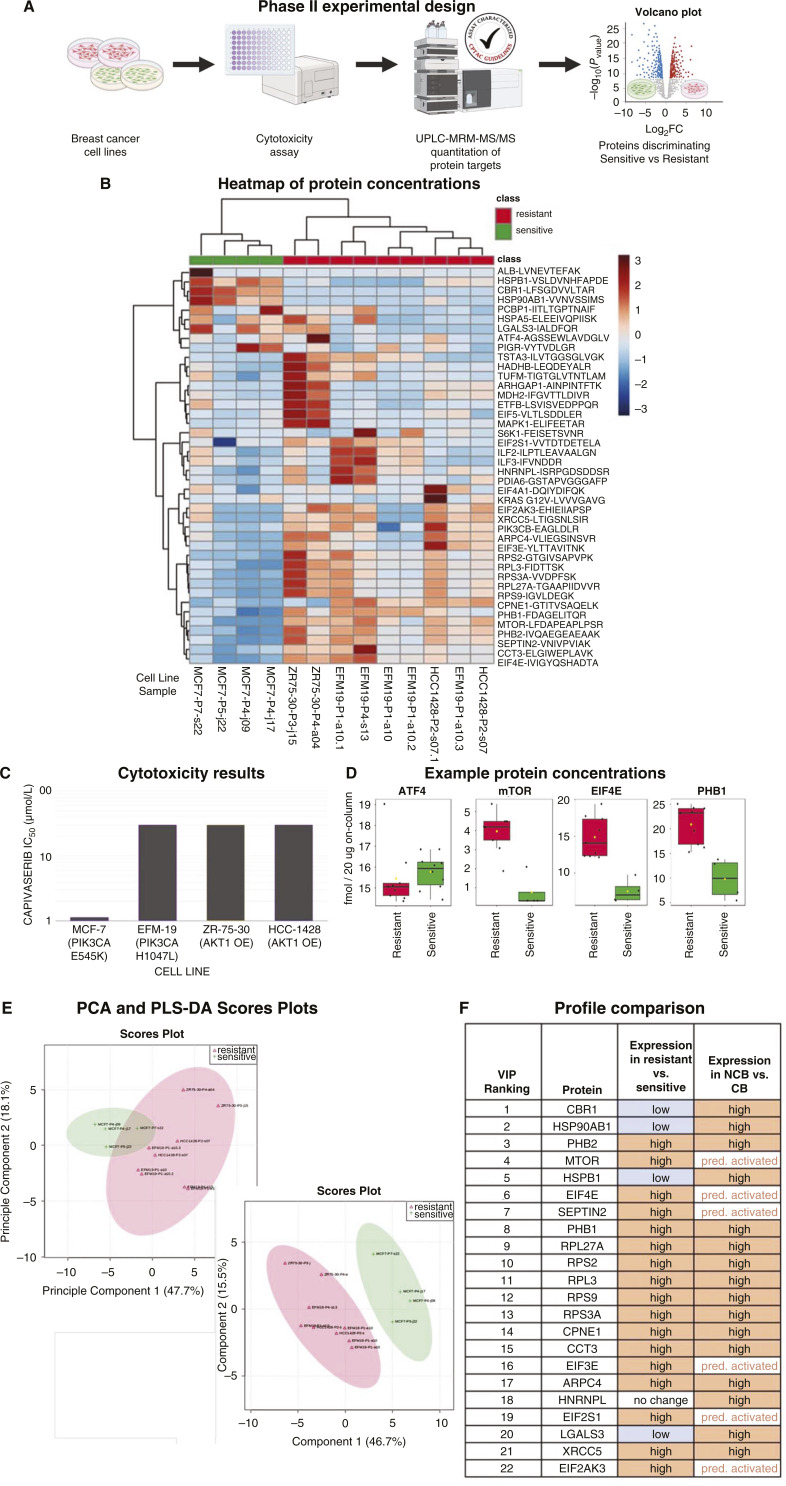
Statistical analysis of targeted protein quantitation in capivasertib-sensitive (green) and capivasertib-resistant (red) HR^+^*PIK3CA*-altered breast cancer cell lines. **A,** Schematic of experimental design for phase II of the study. **B,** Heatmap of protein concentrations quantified by MRM-MS with hierarchical clustering. **C,** Cell line sensitivity to capivasertib, expressed as IC_50_ (**D**) Protein concentrations in capivasertib-sensitive vs. capivasertib-resistant cells (**E**) PCA (left) and PLS-DA plot (right). **F,** Top 22 features ranked by VIP scores, presented with the direction of change in capivasertib-resistant vs. capivasertib cell lines as compared to NCB vs. CB patient tumors.

To further investigate the possible association of the identified proteins and pathways with capivasertib response, we implemented a specially developed multiplexed MRM-MS assay to directly quantify many of the proteins of interest (*n* = 29) and related targets (*n* = 16) with increased precision and reproducibility. The panel was applied to samples from HR^+^ breast cancer cell lines with known activating PIK3CA or AKT1 alterations published in COSMIC (RRID:SCR_002260). Using a standard cytotoxicity assay, MCF-7 was confirmed to be capivasertib-sensitive (IC_50_ < 2 µmol/L), while HCC1428, ZR-75-30, and EFM-19 were found to be capivasertib-resistant (IC_50_ > 10 µmol/L; Fig. 3A). Our CPTAC-validated assays were generally found to be fit-for-purpose in that 42 of the 54 proteins of interest were successfully quantified in most cell line samples. Hierarchical clustering separated sensitive (MCF-7, *n* = 4) from resistant (HCC1428, *n* = 3; ZR-75-30, *n* = 3; EFM-19, *n* = 3) cell lines on the basis of their expression of the proteins of interest (Fig. 3B). When an FDR-adjusted *t* test was applied, assuming unequal variance, three proteins (CBR1, HSP90AB1, HSPB1) were found to be significantly downregulated in the resistant cell lines and 14 were upregulated (FC > 1.5; *P* < 0.05). The proteomic profile observed in the capivasertib-resistant cell lines closely matched the profile observed in the tumors of patients in the NCB group. Of the 22 protein concentrations that best discriminated between sensitive versus resistant cell lines, 17 (77%) showed the same trend detected in the patient study (Fig. 3E; Supplementary Table S2).

**Figure 4:**
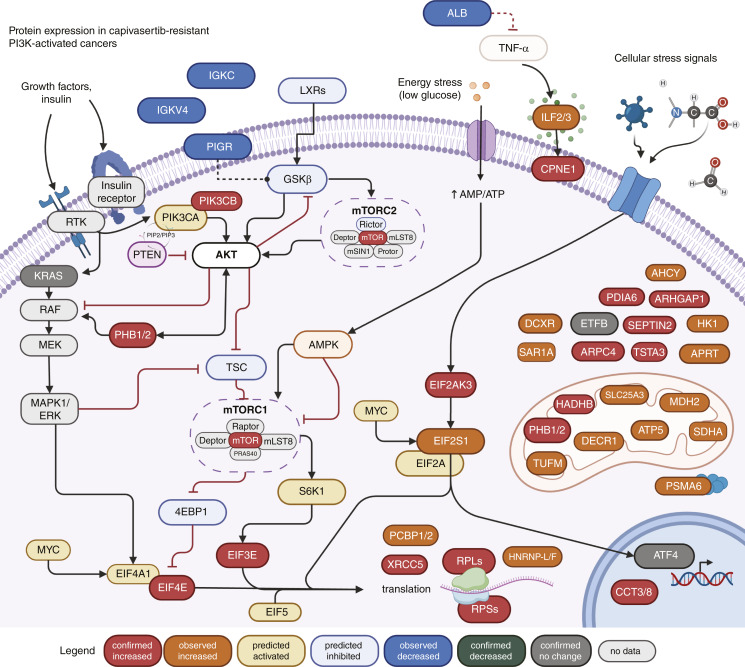
Schematic depicting proteins and pathways differently regulated in capivasertib-resistant cancers, as observed in patient tumors and breast cancer cell lines. Proteins with expression changes confirmed (FC > 1.3; *P* < 0.1) by MRM-MS data in cell lines are shown in red (increased), dark gray (no change), and green (decreased). Proteins with expression data from LFQ in patient tumor samples are shown in dark orange (increased) and dark blue (decreased). Light orange (activated) and light blue (inhibited) indicate proteins with predicted activity changes based on pathway analysis of the LFQ data. Proteins with no expression data are shown in light gray. (Figure Created with BioRender.com.)

When quantified directly, expression of ATF4—a protein whose increased expression directly transduces EIF2-driven translation—is nearly identical between resistant and sensitive cell line samples. This result was further supported by the finding that combination treatment with capivasertib and an eIF2α inhibitor (ISRIB, SelleckChem, S7400) did not yield synergistic effects in the capivasertib-resistant HCC-1428 cell line (Supplementary Fig. S4). While the relationships between the analyzed proteins are complex, the sum of the evidence is consistent with increased downstream mTORC1-driven translation in capivasertib-resistance (Fig. 4). Increased upstream GSKβ/mTORC2-activation might also be associated with capivasertib sensitivity. Given that EIF2 signaling is known to manage the balance between mTORC1 and mTORC2, a role for it cannot be ruled out. Prohibitins (PHB1/2) may be important determinants, as they are known to complex with RAF1 to activate the MEK-ERK pathway (29), which is a known mechanism of resistance to AKT inhibition (30), to increase AKT activity through positive feedback (31), and to support ribosome biogenesis (32).

## Discussion

*AKT* transcript overexpression is frequently observed, and is associated with tumor grade and aggressiveness, irrespective of AKT phosphorylation, in both *PIK3CA*-mutated and *PIK3CA*-wild-type tumors (33, 34). We initially hypothesized that PI3K-altered tumors’ AKT protein concentrations might better correlate with response to the AKT inhibitor capivasertib than genome-only based profiling, but this was not the case within our studied cohort. In addition, despite our expectation of high phospho-AKT concentrations reflecting PI3K pathway activation, few samples showed high phosphorylation levels. Experimental evidence suggests that maximal activation can occur when as little as 5% of the AKT pool is phosphorylated (35), so it is possible that available analytical approaches simply do not quantify such low-abundance modifications with the required precision. However, recent studies have also identified the potential for an uncoupling of AKT activity from *PIK3CA* mutations in some cancers (36, 37). This includes the observation that protein levels of typical markers of pathway activity (e.g., pAKT, pS6, p4EBP1) may be unelevated or even markedly reduced in *PIK3CA*-mutated tumors (36, 38, 39). Understanding the reasons for this disconnect, is an ongoing effort in PI3K-targeted inhibitor development and requires investigation at the proteomic level.

AKT1 and AKT2 protein levels did not differ significantly between the groups. Capivasertib also inhibits AKT3, which has been specifically associated with aggressive hard-to-treat disease and merits further attention in subsequent studies (40). Nonetheless, label-free quantitation data revealed a series of proteins that were differentially expressed between the CB and NCB groups. The profile observed in the CB group could be associated with greater upstream activation of AKT. Alternatively, higher levels of serum albumin and immune-related proteins could also reflect generally better prognosis, better immune infiltration, and greater perfusion of the tumors in the CB group that facilitates treatment. On the other hand, many of the proteins identified as higher in the NCB group have been previously linked to cancer progression, prognosis, and treatment response. For instance, specific subsets of ribosomal proteins have been identified as regulatory factors in various cancers (41, 42). However, the clustering of differential proteins to a handful of interrelated functions, and functional linkages of the proteins to AKT activity, increase the confidence of the findings and support a specific association with response to capivasertib (vs. general prognostic value).

Qiagen IPA analysis pointed to significant activation of EIF2 signaling, EIF4E/S6K and mTOR translational pathways in the NCB group and predicted that Rictor, a key component of mTORC2, may regulate the observed differences through higher activity in the CB group (Supplementary Table S3). As a means of further verifying this profile, we applied a newly validated proteomics panel to reproducibly quantify the proteins of interest from well-characterized cancer cell lines. Remarkably, the observed pattern of differences in protein expression was highly conserved between the patient tumor samples and the cell line model (Fig. 4). The reproducibility of the profile associated with capivasertib resistance across two orthogonal proteomics approaches and two fully independent models lends significant credibility to the proteins’ ability to detect capivasertib sensitivity. Moreover, the targeted assay panel provided key evidence against attributing the observed increase in translational activity specifically to EIF2 signaling, and instead highlighted the importance of downstream mTOR signaling.

Given that no difference in AKT expression or phosphorylation were detected between the CB and NCB groups (both PI3K altered), this study suggests that variability in response to capivasertib monotherapy is likely due to differences in underlying mechanisms of resistance—specifically the downstream mTORC1 activation and increased translation that is evident in the capivasertib-resistant groups. mTORC1-driven resistance to PI3K pathway inhibitors is now well-characterized (43, 44). Previous research points to TSC1/2 or mTOR mutations as a mechanism for mTORC1-driven resistance; mTOR mutation has even been detected and effectively targeted in at least one patient (45). However, additional genetic mechanisms likely exist. Our results provide additional evidence for this pathway of resistance from the tumors of a small cohort of patients in a phase II clinical trial. Moreover, we present some of the first evidence that there is a significant proportion of patients in the genetically preselected intent-to-treat cohort whose tumors demonstrate increased mTORC1 activation years before drug exposure. These patients whose tumors show increased activation downstream from mTORC1 are predictably less likely to benefit from capivasertib monotherapy. The profile can be readily identified with a functional protein quantitation assay from tumor samples collected prior to capivasertib exposure.

## Conclusion

The past almost two decades have seen the rapid emergence of precision medicine, which at first produced evidence that specific genomic variants found in subsets of patient tumors conferred remarkable clinical sensitivity to particular targeted treatments. When the limits of this approach became evident, studies like the WINTHER trial and others demonstrated the added value of transcriptomic data (46). Preclinical and clinical data have since demonstrated that protein levels can diverge from transcriptomic (mRNA) data and can assist in guiding choice of therapy (11, 12). Our workflow in this study uniquely combined targeted and global proteomics technologies, making optimal use of volume-limited clinical samples and demonstrating the ability to obtain useful proteomic data from slide-mounted FFPE collected >7 years prior. We further employed an innovative study model, employing preexisting cell lines to verify findings from clinical samples.

Our data show the value of deeper molecular profiling at the protein level. We found that even in the context of an activating genetic mutation, downstream pathway activity varies sufficiently to alter treatment response. While many cancer cells exploit the PI3K pathway (even in the absence of detectable mutations), some are also primed to subvert AKT inhibition. That the mTORC1-driven profile can be readily observed at the protein level using the developed assay represents a significant opportunity for translation. The panel implemented here may facilitate detection of shifts in mTORC1 activity by downstream proteomic effects, even in the case of unknown or uncharacterized genetic changes, allowing for the correct stratification of patients with tumors whose mutations are not yet well characterize. Similar approaches may also prove valuable to characterize the proteomic profiles associated with sensitivity among tumors that are negative for *known* PI3K pathway alterations—such as was seen in preclinical studies where capivasertib inhibited the growth of cancer cell lines without identifiable PI3K pathway alterations (47). Moreover, the example of EIF2 signaling activation—where molecular changes are rapidly induced without concomitant genetic changes, resulting in uncoupling of the transcriptome and translatome—is another reason to continue prioritizing a proteomics approach.

Although the biological relationships observed in our data are compelling, the certainty of our findings is inherently limited by the limited number of samples analyzed and the heterogeneity of a heavily treated clinical population. CB may not accurately reflect treatment efficacy, especially in the absence of a comparator group (e.g., alternative or placebo treatment) to signal the natural history of disease in this small cohort. The imbalance between primary and metastatic origin for the patient samples also presents an important caveat, though this is partially addressed in our statistical analysis. The validated multiplexed proteomics assay will be a useful tool for future validation efforts, as precise quantitation with internal standards will enable reproducible measurement in larger cohorts and development of reference ranges. Long-term, large-scale randomized prospective studies are required to firmly establish the putative markers’ utility for predicting treatment response in a specified setting. Additional mechanistic studies may also be of value, particularly for pinpointing additional co-targeting strategies (e.g., EIF4 inhibitors).

Further studies will be needed to determine whether the observed profile can be extended to other cancer types, mutational contexts, or combination treatments. Capivasertib is currently being evaluated in phase III trials for activity as part of combination therapies in oncology settings such as prostate cancer, triple-negative breast cancer, and HR^+^ breast cancer (9, 48, 49). Recent results of the CAPItello-291 phase III trial (NCT04305496) demonstrate that treatment with capivasertib + fulvestrant extends PFS in patients with HR^+^/HER2^−^ breast cancers with inadequate response to endocrine therapy. Evidence of treatment benefit was found in the overall population, including patients with and without identifiable AKT pathway alterations (50). As capivasertib moves into earlier stages of treatment and is prescribed to a broader group of patients, we will attempt to validate our findings in contemporaneous tissues, using an approach similar to our other biopsy-driven trials (51).

## Supplementary Material

Supplementary Methods S1 Supplemental Materials and Methods concerning patient samples, cell lines, tissue culture, iMALDI-MS, Nano-LC-Orbitrap-MS, LC-MRM-MS

Supplementary Validation Data S1 Supplementary Validation Data demonstrating the performance of the iMALDI-MS assay

Supplementary Validation Data S2 Quality Assessment of the Global Proteomics Data

Supplementary Figure S1 Individual protein concentrations measured by iMALDI-MS for each sample, comparison of measured PTEN concentration to IHC H-score

Supplementary Figure S2 Individual protein concentrations measured by iMALDI-MS for PTEN vs. IHC H-score

Supplementary Figure S3 Comparison of iMALDI and Global Proteomics results for primary vs metastatic samples

Supplementary Figure S4 Combination treatment of cell lines with capivasertib and an eIF2α inhibitor

Supplementary Table S1 Detailed results of the label-free quantitation

Supplementary Table S2 Proteins expression differences between capivasertib-resistant vs. capivasertib-sensitive cells quantified by validated MRM-MS assays as compared to the profile observed in patient samples

Supplementary Table S3 Detailed results of pathway mapping by Qiagen IPA

## Appendix Gene and protein names.

**Table:**

Gene	Abbreviation	Uniprot	Protein name
*EIF4EBP1*	(p) 4E-BP1	Q13541	Eukaryotic translation initiation factor 4E-binding protein 1
*AHCY*	AdoHcyase	P23526	Adenosylhomocysteinase
*AKT1*	(p)AKT1	P31749	RAC-alpha serine/threonine-protein kinase
*AKT2*	(p)AKT2	P31751	RAC-beta serine/threonine-protein kinase
*ALB*	Albumin	P02768	Albumin
*AMPK*	AMPK α-2	P54646	5′-AMP-activated protein kinase catalytic subunit alpha-2
*APRT*	APRT	P07741	Adenine phosphoribosyltransferase
*ARHGAP1*	p50-RhoGAP	Q07960	Rho GTPase-activating protein 1
*ARPC4*	ARPC4	P59998	Actin-related protein 2/3 complex subunit 4
*ATF4*	ATF4	P18848	Cyclic AMP-dependent transcription factor ATF-4
*CCT3*	TCP-1-gamma	P49368	T-complex protein 1 subunit gamma
*CCT8*	TCP-1-theta	P50990	T-complex protein 1 subunit theta
*CPNE1*	Copine I	Q99829	Copine-1
*DCXR*	XR	Q7Z4W1	L-xylulose reductase
*DECR1*	DECR	Q16698	2,4-dienoyl-CoA reductase [(3E)-enoyl-CoA-producing], mitochondrial
*EIF2A*	eIF-2A	Q9BY44	Eukaryotic translation initiation factor 2A
*EIF2AK3*	PEK, PERK	Q9NZJ5	Eukaryotic translation initiation factor 2-alpha kinase 3
*EIF2S1*	eIF2α	P05198	Eukaryotic translation initiation factor 2 subunit 1
*EIF3E*	eIF3e	P60228	Eukaryotic translation initiation factor 3 subunit E
*EIF4A1*	eIF4A-I	P60842	Eukaryotic initiation factor 4A-I
*EIF4E*	eIF4E	P06730	Eukaryotic translation initiation factor 4E
*EIF5*	eIF-5	P55010	Eukaryotic translation initiation factor 5
*ETFB*	Beta-ETF	P38117	Electron transfer flavoprotein subunit beta
*GSK3B*	GSK-3Beta	P49841	Glycogen synthase kinase-3 beta
*HADHB*	TP-Beta	P55084	Trifunctional enzyme subunit beta, mitochondrial
*HK1*	HK I	P19367	Hexokinase-1
*HNRNP-F*	hnRNP F	P52597	Heterogeneous nuclear ribonucleoprotein F
*HNRNP-L*	hnRNP L	P14866	Heterogeneous nuclear ribonucleoprotein L
*IGKC*	IGKC	P01834	Immunoglobulin kappa constant
*IGKV4*	IGKV4-1	P06312	Immunoglobulin kappa variable 4–1
*ILF2*	ILF2	Q12905	Interleukin enhancer-binding factor 2
*ILF3*	ILF3	Q12906	Interleukin enhancer-binding factor 3
*KRAS*	K-Ras 2	P01116	GTPase KRas
*MAPK1*	MAP kinase 1, ERK2	P28482	Mitogen-activated protein kinase 1
*MDH2*	MDH2	P40926	Malate dehydrogenase, mitochondrial
*MTOR*	mTOR	P42345	Serine/threonine-protein kinase mTOR
*MYC*	c-Myc	P01106	Myc proto-oncogene protein
*PCBP1*	hnRNP E1	Q15365	Poly (rC)-binding protein 1
*PCBP2*	hnRNP E2	Q15366	Poly (rC)-binding protein 2
PDIA6	ERp5	Q15084	Protein disulfide-isomerase A6/Endoplasmic reticulum protein 5
*PHB1*	PHB1	P35232	Prohibitin 1
*PHB2*	PHB2	Q99623	Prohibitin-2
*PIGR*	PIgR	P01833	Polymeric immunoglobulin receptor
*PIK3CA*	PIK3CA, p110α	P42336	Phosphatidylinositol 4,5-bisphosphate 3-kinase catalytic subunit alpha isoform
*PIK3CB*	PIK3C-Beta	P42338	Phosphatidylinositol 4,5-bisphosphate 3-kinase catalytic subunit beta isoform
*PSMA6*	PSMA6	P60900	Proteasome subunit alpha type-6
*PTEN*	PTEN	P60484	Phosphatidylinositol 3,4,5-trisphosphate 3-phosphatase and dual-specificity protein phosphatase PTEN
*RICTOR*	Rictor	Q6R327	Rapamycin-insensitive companion of mTOR
RPS6KB1	(p)S6K1	P23443	Ribosomal protein S6 kinase beta-1
*SDHA*	SDHA	P31040	Succinate dehydrogenase [ubiquinone] flavoprotein subunit, mitochondrial
*SEPTIN2*	Septin-2	Q15019	Septin-2
*SLC25A3*	PTP	Q00325	Phosphate carrier protein, mitochondrial
*TNF*	TNFα	P01375	Tumor necrosis factor alpha
*TSC1*	Hamartin	Q92574	Tuberous sclerosis 1 protein
*TSC2*	Tuberin	P49815	Tuberous sclerosis 2 protein
*TSTA3*	TSTA3	Q13630	GDP-L-fucose synthase
*TUFM*	EF-Tu, P43	P49411	Elongation factor Tu, mitochondrial
*XRCC5*	XRCC5	P13010	X-ray repair cross-complementing protein 5

## References

[1] Millis SZ , Ikeda S, Reddy S, Gatalica Z, Kurzrock R. Landscape of phosphatidylinositol-3-kinase pathway alterations across 19 784 diverse solid tumors. JAMA Oncol 2016;2:1565–73. 27388585 10.1001/jamaoncol.2016.0891

[2] Engelman JA . Targeting PI3K signalling in cancer: opportunities, challenges and limitations. Nat Rev Cancer 2009;9:550–62. 19629070 10.1038/nrc2664

[3] De Luca A , Maiello MR, D’Alessio A, Pergameno M, Normanno N. The RAS/RAF/MEK/ERK and the PI3K/AKT signalling pathways: role in cancer pathogenesis and implications for therapeutic approaches. Expert Opin Ther Targets 2012;16(Suppl 2):S17–27. 10.1517/14728222.2011.639361 22443084

[4] Nitulescu GM , Margina D, Juzenas P, Peng Q, Olaru OT, Saloustros E, . Akt inhibitors in cancer treatment: the long journey from drug discovery to clinical use (Review). Int J Oncol 2016;48:869–85. 26698230 10.3892/ijo.2015.3306 PMC4750533

[5] Turner NC , Oliveira M, Howell SJ, Dalenc F, Cortes J, Gomez Moreno HL, . Capivasertib in hormone receptor–positive advanced breast cancer. N Engl J Med 2023;388:2058–70. 37256976 10.1056/NEJMoa2214131 PMC11335038

[6] Banerji U , Dean EJ, Pérez-Fidalgo JA, Batist G, Bedard PL, You B, . A phase I open-label study to identify a dosing regimen of the pan-AKT inhibitor AZD5363 for evaluation in solid tumors and in *PIK3CA*-mutated breast and gynecologic cancers. Clin Cancer Res 2018;24:2050–9. 29066505 10.1158/1078-0432.CCR-17-2260

[7] Smyth LM , Tamura K, Oliveira M, Ciruelos EM, Mayer IA, Sablin MP, . Capivasertib, an AKT kinase inhibitor, as monotherapy or in combination with fulvestrant in patients with *AKT1*^E17K^-mutant, ER-positive metastatic breast cancer. Clin Cancer Res 2020;26:3947–57. 32312891 10.1158/1078-0432.CCR-19-3953 PMC7415507

[8] Kalinsky K , Hong F, McCourt CK, Sachdev JC, Mitchell EP, Zwiebel JA, . Effect of capivasertib in patients with an AKT1 E17K-mutated tumor: NCI-MATCH subprotocol EAY131-Y nonrandomized trial. JAMA Oncol 2021;7:271–8. 33377972 10.1001/jamaoncol.2020.6741 PMC7774047

[9] Jones RH , Casbard A, Carucci M, Cox C, Butler R, Alchami F, . Fulvestrant plus capivasertib versus placebo after relapse or progression on an aromatase inhibitor in metastatic, oestrogen receptor-positive breast cancer (FAKTION): a multicentre, randomised, controlled, phase 2 trial. Lancet Oncol 2020;21:345–57. 32035020 10.1016/S1470-2045(19)30817-4 PMC7052734

[10] Howell SJ , Casbard A, Carucci M, Ingarfield K, Butler R, Morgan S, . Fulvestrant plus capivasertib versus placebo after relapse or progression on an aromatase inhibitor in metastatic, oestrogen receptor-positive, HER2-negative breast cancer (FAKTION): overall survival, updated progression-free survival, and expanded biomarker analysis from a randomised, phase 2 trial. Lancet Oncol 2022;23:851–64. 35671774 10.1016/S1470-2045(22)00284-4 PMC9630162

[11] Li L , Wei Y, To C, Zhu CQ, Tong J, Pham NA, . Integrated omic analysis of lung cancer reveals metabolism proteome signatures with prognostic impact. Nat Commun 2014;5:5469. 25429762 10.1038/ncomms6469

[12] Roumeliotis TI , Williams SP, Goncalves E, Alsinet C, Del Castillo Velasco-Herrera M, Aben N, . Genomic determinants of protein abundance variation in colorectal cancer cells. Cell Rep 2017;20:2201–14. 28854368 10.1016/j.celrep.2017.08.010 PMC5583477

[13] Popp R , Li H, LeBlanc A, Mohammed Y, Aguilar-Mahecha A, Chambers AG, . Immuno-matrix-assisted laser desorption/ionization assays for quantifying AKT1 and AKT2 in breast and colorectal cancer cell lines and tumors. Anal Chem 2017;89:10592–600. 28853539 10.1021/acs.analchem.7b02934

[14] Domanski D , Murphy LC, Borchers CH. Assay development for the determination of phosphorylation stoichiometry using multiple reaction monitoring methods with and without phosphatase treatment: application to breast cancer signaling pathways. Anal Chem 2010;82:5610–20. 20524616 10.1021/ac1005553 PMC2909760

[15] Froehlich BC , Popp R, Sobsey CA, Ibrahim S, LeBlanc A, Mohammed Y, . A multiplexed, automated immuno-matrix assisted laser desorption/ionization mass spectrometry assay for simultaneous and precise quantitation of PTEN and p110α in cell lines and tumor tissues. Analyst 2021;146:6566–75. 34585690 10.1039/d1an00165e

[16] Whiteaker JR , Halusa GN, Hoofnagle AN, Sharma V, MacLean B, Yan P, . CPTAC assay portal: a repository of targeted proteomic assays. Nat Methods 2014;11:703–4. 24972168 10.1038/nmeth.3002 PMC4113142

[17] Pang Z , Chong J, Zhou G, de Lima Morais DA, Chang L, Barrette M, . MetaboAnalyst 5.0: narrowing the gap between raw spectra and functional insights. Nucleic Acids Res 2021;49:W388–96. 34019663 10.1093/nar/gkab382 PMC8265181

[18] Szklarczyk D , Gable AL, Nastou KC, Lyon D, Kirsch R, Pyysalo S, . The STRING database in 2021: customizable protein–protein networks, and functional characterization of user-uploaded gene/measurement sets. Nucleic Acids Res 2021;49:D605–12. 33237311 10.1093/nar/gkaa1074 PMC7779004

[19] Doncheva NT , Morris JH, Gorodkin J, Jensen LJ. Cytoscape StringApp: network analysis and visualization of proteomics data. J Proteome Res 2019;18:623–32. 30450911 10.1021/acs.jproteome.8b00702 PMC6800166

[20] Fiala O , Pesek M, Finek J, Racek J, Minarik M, Benesova L, . Serum albumin is a strong predictor of survival in patients with advanced-stage non-small cell lung cancer treated with erlotinib. Neoplasma 2016;63:471–6. 26952513 10.4149/318_151001N512

[21] Dalmiglio C , Brilli L, Campanile M, Ciuoli C, Cartocci A, Castagna MG. CONUT score: a new tool for predicting prognosis in patients with advanced thyroid cancer treated with TKI. Cancers (Basel) 2022;14:724. 35158991 10.3390/cancers14030724 PMC8833681

[22] Jones DT , Ganeshaguru K, Anderson RJ, Jackson TR, Bruckdorfer KR, Low SY, . Albumin activates the AKT signaling pathway and protects B-chronic lymphocytic leukemia cells from chlorambucil- and radiation-induced apoptosis. Blood 2003;101:3174–80. 12480711 10.1182/blood-2002-07-2143

[23] Tey SK , Wong SWK, Chan JYT, Mao X, Ng TH, Yeung CLS, . Patient pIgR-enriched extracellular vesicles drive cancer stemness, tumorigenesis and metastasis in hepatocellular carcinoma. J Hepatol 2022;76:883–95. 34922977 10.1016/j.jhep.2021.12.005

[24] Dianat-Moghadam H , Khalili M, Keshavarz M, Azizi M, Hamishehkar H, Rahbarghazi R, . Modulation of LXR signaling altered the dynamic activity of human colon adenocarcinoma cancer stem cells *in vitro*. Cancer Cell Int 2021;21:100. 33568147 10.1186/s12935-021-01803-4 PMC7877018

[25] Holcik M . Could the eIF2alpha-independent translation be the achilles heel of cancer? Front Oncol 2015;5:264. 26636041 10.3389/fonc.2015.00264 PMC4659918

[26] Rajesh K , Krishnamoorthy J, Kazimierczak U, Tenkerian C, Papadakis AI, Wang S, . Phosphorylation of the translation initiation factor eIF2α at serine 51 determines the cell fate decisions of Akt in response to oxidative stress. Cell Death Dis 2015;6:e1591. 25590801 10.1038/cddis.2014.554 PMC4669752

[27] Ribas R , Pancholi S, Guest SK, Marangoni E, Gao Q, Thuleau A, . AKT antagonist AZD5363 influences estrogen receptor function in endocrine-resistant breast cancer and synergizes with fulvestrant (ICI182780) *in vivo*. Mol Cancer Ther 2015;14:2035–48. 26116361 10.1158/1535-7163.MCT-15-0143

[28] Chen X , Dai X, Zou P, Chen W, Rajamanickam V, Feng C, . Curcuminoid EF24 enhances the anti-tumour activity of Akt inhibitor MK-2206 through ROS-mediated endoplasmic reticulum stress and mitochondrial dysfunction in gastric cancer. Br J Pharmacol 2017;174:1131–46. 28255993 10.1111/bph.13765 PMC5406301

[29] Bavelloni A , Piazzi M, Faenza I, Raffini M, D’Angelo A, Cattini L, . Prohibitin 2 represents a novel nuclear AKT substrate during all-trans retinoic acid-induced differentiation of acute promyelocytic leukemia cells. FASEB J 2014;28:2009–19. 24522204 10.1096/fj.13-244368

[30] McCubrey JA , Steelman LS, Chappell WH, Abrams SL, Franklin RA, Montalto G, . Ras/Raf/MEK/ERK and PI3K/PTEN/Akt/mTOR cascade inhibitors: how mutations can result in therapy resistance and how to overcome resistance. Oncotarget 2012;3:1068–111. 23085539 10.18632/oncotarget.659 PMC3717945

[31] Bao F , Hao P, An S, Yang Y, Liu Y, Hao Q, . Akt scaffold proteins: the key to controlling specificity of Akt signaling. Am J Physiol Cell Physiol 2021;321:C429–2. 34161152 10.1152/ajpcell.00146.2020

[32] Zhou Z , Ai H, Li K, Yao X, Zhu W, Liu L, . Prohibitin 2 localizes in nucleolus to regulate ribosomal RNA transcription and facilitate cell proliferation in RD cells. Sci Rep 2018;8:1479. 29367618 10.1038/s41598-018-19917-7 PMC5784149

[33] Cizkova M , Vacher S, Meseure D, Trassard M, Susini A, Mlcuchova D, . PIK3R1 underexpression is an independent prognostic marker in breast cancer. BMC Cancer 2013;13:545. 24229379 10.1186/1471-2407-13-545 PMC4225603

[34] Kim SH , Seung BJ, Cho SH, Lim HY, Bae MK, Sur JH. Dysregulation of PI3K/Akt/PTEN pathway in canine mammary tumor. Animals (Basel) 2021;11:2079. 34359206 10.3390/ani11072079 PMC8300234

[35] Hoehn KL , Hohnen-Behrens C, Cederberg A, Wu LE, Turner N, Yuasa T, . IRS1-independent defects define major nodes of insulin resistance. Cell Metab 2008;7:421–33. 18460333 10.1016/j.cmet.2008.04.005 PMC2443409

[36] Vasudevan KM , Barbie DA, Davies MA, Rabinovsky R, McNear CJ, Kim JJ, . AKT-independent signaling downstream of oncogenic PIK3CA mutations in human cancer. Cancer Cell 2009;16:21–32. 19573809 10.1016/j.ccr.2009.04.012 PMC2752826

[37] Faes S , Dormond O. PI3K and AKT: unfaithful partners in cancer. Int J Mol Sci 2015;16:21138–52. 26404259 10.3390/ijms160921138 PMC4613246

[38] Stemke-Hale K , Gonzalez-Angulo AM, Lluch A, Neve RM, Kuo WL, Davies M, . An integrative genomic and proteomic analysis of PIK3CA, PTEN, and AKT mutations in breast cancer. Cancer Res 2008;68:6084–91. 18676830 10.1158/0008-5472.CAN-07-6854 PMC2680495

[39] Cancer Genome Atlas Network . Comprehensive molecular portraits of human breast tumours. Nature 2012;490:61–70. 23000897 10.1038/nature11412 PMC3465532

[40] O’Hurley G , Daly E, O’Grady A, Cummins R, Quinn C, Flanagan L, . Investigation of molecular alterations of AKT-3 in triple-negative breast cancer. Histopathology 2014;64:660–70. 24138071 10.1111/his.12313

[41] Lin Z , Peng R, Sun Y, Zhang L, Zhang Z. Identification of ribosomal protein family in triple-negative breast cancer by bioinformatics analysis. Biosci Rep 2021;41:BSR20200869. 33305312 10.1042/BSR20200869 PMC7789804

[42] Fang E , Zhang X. Identification of breast cancer hub genes and analysis of prognostic values using integrated bioinformatics analysis. Cancer Biomark 2017;21:373–81. 29081411 10.3233/CBM-170550 PMC13078278

[43] Andrikopoulou A , Chatzinikolaou S, Panourgias E, Kaparelou M, Liontos M, Dimopoulos MA, . The emerging role of capivasertib in breast cancer. Breast 2022;63:157–67. 35398754 10.1016/j.breast.2022.03.018 PMC9011110

[44] Dunn S , Eberlein C, Yu J, Gris-Oliver A, Ong SH, Yelland U, . AKT-mTORC1 reactivation is the dominant resistance driver for PI3Kβ/AKT inhibitors in PTEN-null breast cancer and can be overcome by combining with Mcl-1 inhibitors. Oncogene 2022;41:5046–60. 36241868 10.1038/s41388-022-02482-9 PMC9652152

[45] Coleman N , Subbiah V, Pant S, Patel K, Roy-Chowdhuri S, Yedururi S, . Emergence of mTOR mutation as an acquired resistance mechanism to AKT inhibition, and subsequent response to mTORC1/2 inhibition. NPJ Precis Oncol 2021;5:99. 34853384 10.1038/s41698-021-00240-w PMC8636467

[46] Rodon J , Soria JC, Berger R, Miller WH, Rubin E, Kugel A, . Genomic and transcriptomic profiling expands precision cancer medicine: the WINTHER trial. Nat Med 2019;25:751–8. 31011205 10.1038/s41591-019-0424-4 PMC6599610

[47] Davies BR , Greenwood H, Dudley P, Crafter C, Yu D-H, Zhang J, . Preclinical pharmacology of AZD5363, an inhibitor of AKT: pharmacodynamics, antitumor activity, and correlation of monotherapy activity with genetic background. Mol Cancer Ther 2012;11:873–87. 22294718 10.1158/1535-7163.MCT-11-0824-T

[48] Schmid P , Abraham J, Chan S, Wheatley D, Brunt AM, Nemsadze G, . Capivasertib plus paclitaxel versus placebo plus paclitaxel as first-line therapy for metastatic triple-negative breast cancer: the PAKT trial. J Clin Oncol 2020;38:423–33. 31841354 10.1200/JCO.19.00368

[49] Smyth LM , Batist G, Meric-Bernstam F, Kabos P, Spanggaard I, Lluch A, . Selective AKT kinase inhibitor capivasertib in combination with fulvestrant in PTEN-mutant ER-positive metastatic breast cancer. NPJ Breast Cancer 2021;7:44. 33863913 10.1038/s41523-021-00251-7 PMC8052445

[50] Turner N , Oliveira M, Howell SJ, Dalenc F, Cortés J, Gomez H, . Abstract GS3-04: GS3-04 capivasertib and fulvestrant for patients with aromatase inhibitor-resistant hormone receptor-positive/human epidermal growth factor receptor 2-negative advanced breast cancer: results from the phase III CAPItello-291 trial. Cancer Res 2023;83(5_Suppl):GS3–04.

[51] Gambaro K , Marques M, McNamara S, Couetoux du Tertre M, Diaz Z, Hoffert C, . Copy number and transcriptome alterations associated with metastatic lesion response to treatment in colorectal cancer. Clin Transl Med 2021;11:e401. 33931971 10.1002/ctm2.401 PMC8087915

