# Supplementary material for: mTORC1-Driven Protein Translation Correlates with Clinical Benefit of Capivasertib within a Genetically Preselected Cohort of PIK3CA-Altered Tumors
Source: Cancer Res Commun. 2024 Aug 13;4(8):2058–74. doi: 10.1158/2767-9764.CRC-24-0113 (PMC11320025; doi:10.1158/2767-9764.CRC-24-0113)
Supplement: Supplementary Methods S1 — Supplemental Materials and Methods concerning patient samples, cell lines, tissue culture, iMALDI-MS, Nano-LC-Orbitrap-MS, LC-MRM-MS [file crc-24-0113_supplementary_methods_s1_suppsm1.pdf]

Supplemental Methods S1

Patient Samples

Research ethics approval for this study was granted by the Research Ethics Committee at the Jewish General Hospital in Montreal, Quebec, Canada (Project #2018-663, 17-004). Anonymized patient tumour samples were obtained from AstraZeneca's multi-centre clinical trial of AZD5363 (NCT01226316) (1). The sample set for the current study was drawn from the Part C dose-expansion cohort, which included patients with breast cancer whose tumour pathology was either estrogen-receptor positive (ER+) or human epidermal growth factor receptor 2-positive (HER2+) breast cancer, as well as patients with gynecological (ovarian, cervical, or endometrial) cancers for whom no standard therapy was effective.

For this cohort, eligibility was restricted to patients whose tumours contained known activating *PIK3CA* mutation(s), as detected by PCR-based approaches during screening (local testing). As described in Banerji et al. (1), exclusion criteria included prior treatment with PI3K inhibitors or glucose metabolism abnormalities. The enrolled patients received capivasertib 480 mg twice-daily for 4 days followed by 3 days off, repeated in 21-day cycles. Tumour volume was measured according to RECIST 1.1 criteria within 28 days of the start of treatment and at specified timepoints after the start of treatment (i.e., weeks 6, 12, 18, 24, etc.). RECIST v1.1 criteria were used to categorize target lesion response into complete response (CR), partial response (PR, ≥30% decrease in volume), stable disease (SD, volume ±<30%), or progressive disease (PD, ≥30% increase in volume) (1). Treatment was continued as tolerated, until either withdrawal of consent or evidence of disease progression.

Patient tumour samples were obtained at the time of screening for genetic/histological assessment and were stored at room temperature as 4 µm-thick FFPE tumour tissue slices mounted to glass slides. A subset of 2 slides per tumour for each of 24 tumour samples was selected for our study, of which a total of 23 slides, representing 16 tumours, yielded sufficient material (≥25 µg total protein/slide) for the planned analyses. For each tumour, standardized, anonymized response data was used to classify patient drug response based on progression-free survival (PFS; target lesion meeting criteria for SD, PR, or CR). "Clinical Benefit" (CB) was defined as PFS for a minimum of 12 weeks after starting capivasertib. "No Clinical Benefit" (NCB) was defined as a target lesion meeting the criteria for progressive disease (PD) observed within 12 weeks of starting capivasertib.

Cell Lines & Tissue Culture

Materials. Capivasertib was obtained from MedChemExpress, dissolved in DMSO at a concentration of 30 mM, and stored at -20ºC for a maximum of 3 months. Well-characterized hormone-receptor positive, PIK3CA-altered breast cancer cell lines were selected for analysis: HCC-1428 (CRL-2327, ATCC), EFM-19 (ACC 231, DSMZ), ZR-75-30 and MCF-7 (gifted from Dr. Basik lab, Segal Cancer Centre). Information about mutational status and expression of AKT1, AKT2, AKT3, PIK3CA, PIK3CB, PTEN, ERBB2 was obtained exclusively from the Cosmic Cell Lines Project (v97, released 29 Nov 2022, https://cancer.sanger.ac.uk/cell_lines) (2).

Cell line	Medium	Breast cancer type & relevant mutations
**EFM-19**<br>(ACC 231, DSMZ)	RPMI-1640 + 15% h.i. FBS + 1% P/S	HR+, HER2-overexpressing<br>PIK3CA mutation (H1047L)<br>PTEN wild-type
**MCF-7**<br>(Basik lab)	RPMI-1640 +10% h.i. FBS + 1% P/S	HR+, HER2 -<br>PIK3CA mutation (E545K)<br>PTEN wild-type
**HCC-1428**<br>(CRL-2327 ATCC)	RPMI-1640 +10% h.i. FBS + 1% P/S	HR+, HER2 -<br>AKT1 overexpressing (ploidy 3.52)<br>PTEN wild-type
**ZR-75-30**<br>(Basik lab)	RPMI-1640 +10% h.i. FBS + 1% P/S	HR+, HER2 overexpressing<br>AKT1 overexpressing<br>PTEN wild-type

Cell culture maintenance. All cell lines were cultured in RPMI-1640 with 10% or 15% fetal bovine serum (FBS), with 1% penicillin and streptomycin (10,000 U/mL, ThermoFisher Scientific). Cell lines were tested for mycoplasma contamination and treated prophylactically after long-term storage with 14-day incubation with 0.1% ciprofloxacin (Bioworld ciprofloxacin hydrochloride, 10 mg/mL, 1000X) prior to expansion (3). Each cell line was

maintained at 37°C in a 5% CO2 humidified incubator. The medium was exchanged every 2-5 days, as required. Cell cultures were maintained at less than 85% confluency and passaged as needed using 0.25 % trypsin + EDTA, for no more than 8 passages.

Proteomics sampling. Samples for proteomics were collected prior to capivasertib exposure, in at least triplicate, with replicates for a given cell line taken from different passages where possible. At the time of collection, adhered cells were gently lysed using 0.25 % trypsin + EDTA, washed with sterile PBS, then pelleted and immediately stored at -80ºC until extraction.

Capivasertib cytotoxicity assay. A standard alamarBlue™-based cell viability assay was used to assess cell lines' sensitivity to capivasertib. Cells were seeded onto 96-well plates at least 24 hours prior to treatment. The number of cells seeded for cytotoxicity assays (~2000-10,000 cells per well) was optimized based on the observed doubling time of each individual cell line to maximize signal without reaching confluency. Drugs were prepared in DMSO. On Day 0, medium was exchanged for medium with capivasertib (AZD5363), with a final concentration of 0 to 30 uM drug and 0.3% DMSO in the medium. After 72 hours of incubation with treatment, medium was replaced with 100 uL alamarBlue™ (Invitrogen) and incubated for approximately 30 minutes. An EnSpire® Multimode Plate Reader (PerkinElmer) was used to measure fluorescence with an excitation wavelength at 530–560 nm and an emission wavelength at 590 nm in the interior 60 wells of the plate. Net growth inhibition for each drug level was calculated as: (mean DMSO reading - mean experimental reading)/(mean DMSO reading). Inhibitory concentrations (IC_{50} and IC_{15}) were determined on the basis of at least 3 fully independent replicates.

iMALDI-MS Method

Protein extraction & total protein quantitation. Solutions were prepared using LC-MS grade water and solvents, and analytical grade reagents. Samples were extracted using xylene deparaffinization followed by stepwise ethanol rehydration, high-temperature incubation in sample extraction buffer (2% sodium deoxycholate in 50 mMol Tris-HCl, pH 8, 20 minutes, 99°C) to break formalin crosslinks, sonication (25% power, 15 sec, 2 rounds), and an additional incubation (2 hours, 80°C). The concentration of total protein in each sample was then quantified using a Pierce bicinchoninic acid (BCA) protein assay kit (Thermo Scientific, Cat # 23225) with a ThermoFisher Multiskan GO spectrophotometer.

Sample preparation for iMALDI-MS of AKT. A published workflow for quantitation of AKT by immuno-MALDI-MS was implemented with automation, as previously described (4-6). The assay is compatible with fresh frozen or formalin-fixed paraffin-embedded (FFPE) tissue samples and can be performed on as little as 100-200 ug of tissue (~10 ug of total protein per analyte). Briefly, aliquots of 40 µg of total protein were diluted to 0.1 µg/µL total protein and prepared in an automated fashion on an Agilent Bravo Liquid Handling Robot. The samples were further divided into aliquots of 100 µL (10 µg total protein) each for independent quantitation of surrogate peptides for AKT1 and AKT2 with and without phosphatase treatment, as described in Domanski et al (7). A standard calibration curve was prepared by spiking known quantities (20, 10, 5, 2.5, 1.25, 0.63, 0.32, 0 fmol) of unlabeled standard peptide (AKT1: ^{466}RPHFPQFSYSASGTA^{480}, AKT2: ^{468}THFPQFSYSASIRE^{481}) into 10 ug of bovine serum albumin at 0.1 µg/µL.

Tryptic digestion & dephosphorylation. Each sample or calibration standard was then denatured and reduced with 10 µL sodium deoxycholate denaturing mix (20 mM TrisHCl, pH 8.4, 0.74 mM TCEP, 10% DOC) at 60ºC for 30 minutes. Samples were then brought to room temperature before alkylation with 10 µL 0.74 mM iodoacetamide (IAA) for 30 minutes while protected from light, and quenching of any remaining IAA with 10 µL 0.74 mM dithiothreitol (DTT). Digestion was performed with trypsin (Worthington, TPCK Treated, 97% purity) dissolved in 1mM HCl, added at a substrate-to-enzyme ratio of 2:1, and allowed to incubate for 1 hour at 37°C. The digest was then chilled on ice and quenched with Nα-Tosyl-L-lysine chloromethyl ketone hydrochloride (TLCK) in a 2-fold molar ratio over trypsin. The dephospohorylation step was performed on selected samples by incubating with or without alkaline phosphatase (1U/µg total protein) for 2 hours at 37°C. The relevant stable isotope-labeled internal standard peptide (AKT1:^{466}<u>R</u>PHFPQFSYSASGTA^{480}, AKT2: ^{468}THFPQFSYSASI<u>R</u>E^{481}) was then added to each aliquot prior to immunoenrichment.

Patient tumour analysis workflow

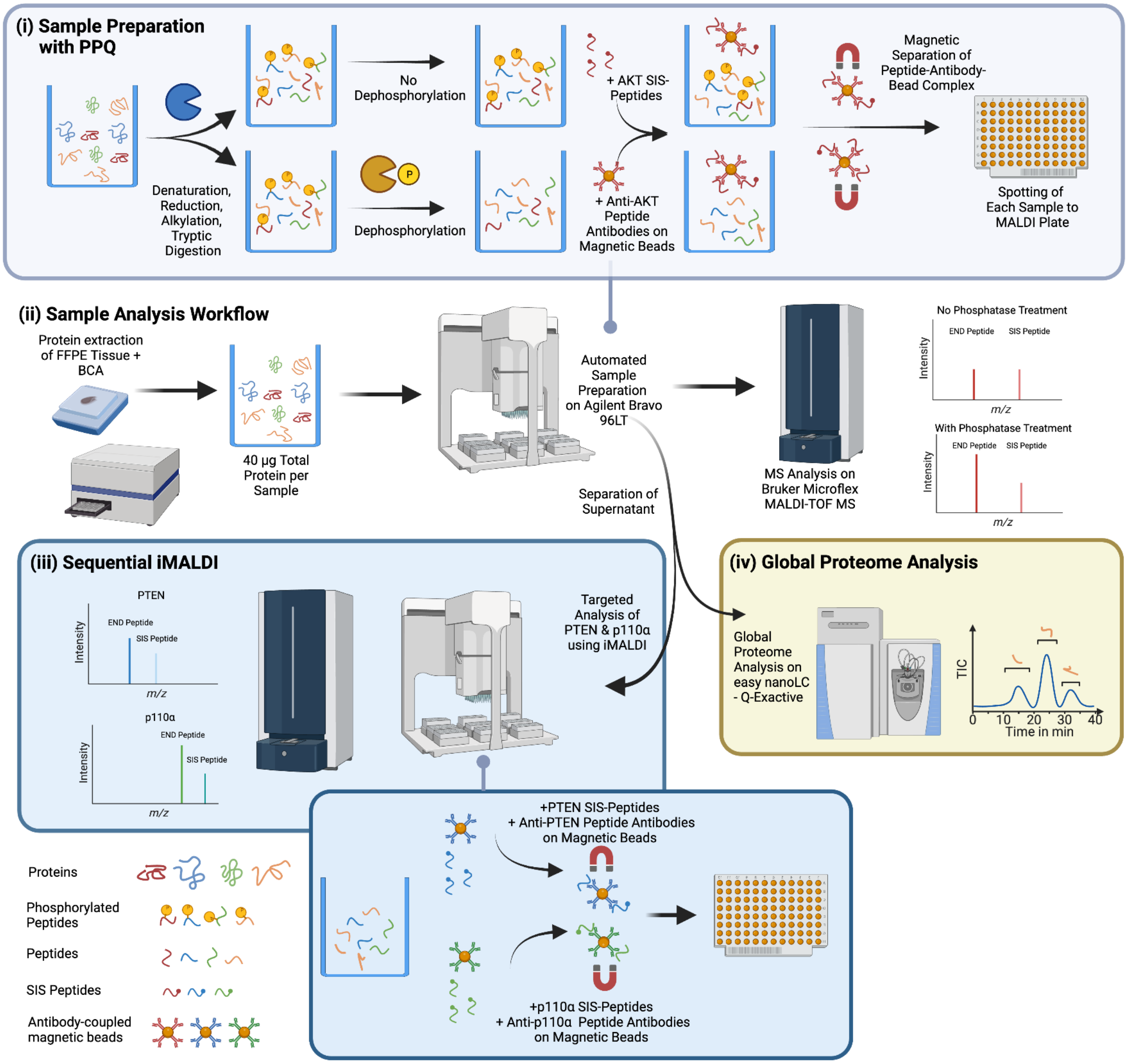

Figure 1. Sample analysis workflow for FFPE tumour samples.

(i) Proteins were extracted from samples and digested with trypsin. Phosphorylation stoichometry was assessed using phosphatase-based phosphopeptide quantitation (PPQ). Following the addition of stable isotope labeled standard (SIS) peptides, AKT1/AKT2 were immuno-enriched together with their internal standards and quantified by iMALDI against an external calibration curve. (ii) Using a novel sample analysis workflow, supernatants from the AKT enrichment step were retained for subsequent analyses. (iii) PTEN + PI3K p110α were quantified from sequential iMALDI of the supernatants. (iv) A small aliquot of supernatant was reserved for label -free quantitation (LFQ) of the global proteome.

Immunoenrichment & spotting. Immunoenrichment was achieved by tumbling samples overnight at 4ºC with anti-peptide antibodies (Signatope GmbH) coupled to magnetic beads (ThermoFisher Protein G Dynabeads). Beads were washed with ammonium bicarbonate (5 mM), 15% ACN/phosphate-buffered saline, and 15% ACN/ammonium bicarbonate (4.25 mM), prior to spotting on a MALDI target. HCCA MALDI matrix consisting of 3 mg/mL cyano-4-hydroxycinnamic acid, 7 mM ammonium citrate, 70% ACN, 0.15% TFA was applied to the dried spot, which was then washed with 7 mM ammonium citrate. The spotted plate was stored at room temperature until analysis.

AKT MALDI-MS Acquisition & Data Analysis. Mass spectra were acquired on a Bruker Microflex™ LRF benchtop MALDI-TOF-MS. Instrument settings were optimized in the linear mode to maximize signal intensity. Data analysis was performed in Flexanalysis 3.4 using Savitsky-Golay smoothing, baseline subtraction, and automated peak picking. Concatenation of peak intensity measurements from spectra was performed with MS-VIS (mass-spectrum.com) (8). Linear range and precision were evaluated using a calibration curve of synthetic peptides spiked. The calibration curve was generated using a linear regression with a $1/x^2$ weighting. The performance of the optimized iMALDI-MS assays was confirmed with quality control samples (QCs) prior to analysis of the clinical samples.

Statistical Analysis. Descriptive statistics and protein concentration data were analyzed in Microsoft Excel. Group differences were assessed using the common t-test and non-parametric tests to address the possibility of non-normal distributions in the protein concentration data. Boxplots were generated with the BoxPlotR webserver (9).

Supernatant Analysis. The AKT-depleted supernatant from this step was retained; a portion was reserved for global proteome analysis by nano-LC-Orbitrap MS, and the remainder was enriched for PTEN and PI3K p110α using anti-PTEN and PI3K p110α anti-peptide antibodies. The PTEN and PI3K p110α antibody-coupled beads were spotted on the MALDI target and quantitated using our previously-validated iMALDI method (19). The peptide used for PI3K p110α quantitation (^{503}EAGFSYSHAGLSNR^{516}) did not overlap with the sequences affected by the *PIK3CA* mutations detected in this patient group.

Nano-LC-Orbitrap-MS

Sample preparation for Nano-LC-Orbitrap MS analysis. A 2-µL aliquot of the AKT-depleted supernatant from each sample digest's immunoenrichment, corresponding to 117.5 ng of total protein digest, was reserved from the iMALDI workflow for label-free quantitation of non-targeted peptides. Samples were prepared for LC-MS using custom StageTips, consisting of a 200 µL pipette tip loaded with Oligo R3 material and C18 disk, for desalting. Tips were activated with 100 % ACN and equilibrated with 0.1 % trifluoroacetic acid (TFA), prior to loading the supernatant samples (with flow-through reloaded 2x), which were washed with 0.1 % TFA and eluted with 70 % ACN, 0.1 % TFA. Samples were then dried under vacuum and resuspended in 6 µL of 0.1% formic acid (batch 1) or 10 µL of 0.1% formic acid (batch 2). Five (5) µL of each sample was injected in Batch 1, resulting in 107 ng of total protein digest injected on-column. Ten (10) µL of each sample was injected in Batch 2, resulting in ~117 ng of total protein digest injected on-column.

Online nano-LC. Online liquid chromatography was performed on an Easy-nLC 1200 (Thermo Fisher Scientific, Waltham, MA, USA) coupled to a ThermoFisher Scientific Q-Exactive Plus (Thermo Fisher Scientific) mass spectrometer that was operated with a Nanospray Flex ion source (Thermo Fisher Scientific). The LC system was equipped with an AcclaimPepMap 100 C18 pre-column (Thermo Fisher Scientific, 3 µm particle size, 75 µm inner diameter × 2 cm length) and a nanoscale analytical column (Thermo Fisher Scientific, AcclaimPepMap 100 C18 main column, 2 µm particle size, 75 µm inner diameter x 25 cm length). Chromatography was performed with mobile phase A: 0.1% formic acid and mobile phase B: 84% acetonitrile, 0.1% formic acid. The 78-minute method, optimized for complex samples, uses a 300 nL/minute flow rate at 20 ºC and incorporates a 50-minute gradient (3 to 17% B for 30 minutes, 17 to 40% B for 20 minutes).

Orbitrap-MS data acquisition. Mass spectra were acquired on the Q-Exactive using a data-dependent acquisition (DDA) method, where the 15 most abundant precursor ions (charge states: 2+ to 4+) were selected for MS/MS fragmentation. Full MS scans were acquired over the mass range from m/z 350 to m/z 1500 at 70,000 resolution

using automatic gain control (AGC) target value of 1 x 10^6 and a maximum injection time of 50 ms. MS2 spectra were acquired with an isolation width of m/z 1.2, an AGC target value of 2 x 10^4, and a maximum injection time of 64 ms, at a resolution of 17,500. Fragmentation was performed using higher energy collisional dissociation (HCD) with a normalized collision energy of 28. Dynamic exclusion was set at 40 seconds.

Data analysis. MS raw data were processed using Proteome Discoverer™ 2.4 (PD, Thermo Scientific). Database searches were performed using SequestHT and a human Swissprot database (January 2019; 20,414 target entries), with trypsin as the enzyme and a maximum of 1 missed cleavage. Carbamidomethylation of cysteine (+57.021 Da) was set as a fixed modification and oxidation of methionine (+15.995 Da) as a variable modification. Mass tolerances were set to 10 ppm for precursor ions and 0.02 Da for product ions. Percolator algorithm was used to calculate posterior error probabilities and the data was filtered to a false discovery rate (FDR) of <1% on the peptide and protein levels. Label-free quantitation (LFQ) was performed using the Minora feature-detector node and applying low-abundance resampling imputation which replaces missing values randomly with values from the lower 5% of detected values. Samples were normalized based on the total summed protein intensities to correct for differences in sample loading. For each protein, the obtained abundances were also scaled to reach a fixed total value when summed by feature while maintaining the ratio observed in the samples -- this facilitates comparisons by representing different features on the same scale. Where applicable, scaled abundances of the two technical replicate measurements were averaged. Scaled abundances for proteins quantified in datasets SN1 and SN2 were then combined. Only proteins that were quantified with at least one peptide unique to that protein were included for the quantitative comparison. The scaled, normalized abundances were exported to Microsoft Excel, and further filtered by selecting protein IDs with "high" confidence (i.e., <1% chance of FDR) and at least 2 peptides unique to that protein.

Statistical analysis. Multivariate statistical analysis was performed using the MetaboAnalyst webserver (www.metaboanalyst.ca) **(10)**. Features with >35% missing values across all samples were excluded from statistical analysis. Samples were re-normalized by sum following removal of incomplete variables. "Auto" data scaling, which expresses each variable measurement as the standard deviation from the variable's mean, was used to ensure equal weighting of features. MetaboAnalyst generated volcano plots, Principle Component Analysis, Partial Least Squares Discriminant Analysis, Variable Importance in the Projection (VIP) scores, heatmaps, and hierarchical clustering based on the normalized data. StringDB was applied for network analysis, to identify clusters of related proteins, and to identify relevant publications that reference similar clusters of proteins **(11)**. Cytoscape was used to visualize the regulation of proteins within the network **(12)**. QIAGEN Ingenuity Pathway Analysis (QIAGEN IPA) software was used to perform pathway mapping of the remaining proteins based on the scaled LFQ data from Proteome Discoverer.

LC-MRM-MS Assays

Assay development & validation. Targeted multiplexed UPLC-MRM-MS assays were developed and optimized for 54 proteins of interest using synthetic proteotypic peptides for calibration and corresponding stable-isotope labelled standard peptides for quantitation (Sobsey et al, in preparation). Assays were characterized in accordance with the NCI's CPTAC guidelines; validation data is publicly available in the CPTAC portal (http://assays.cancer.gov/) (13). The assays have been demonstrated as fit-for-purpose under Tier II applications (pre-clinical research) (14).

CPTAC ID	PEPTIDE SEQUENCE	URL
non-CPTAC-6077	FFAGIVWQHVYEK	https://assays.cancer.gov/non-CPTAC-6077
non-CPTAC-6078	YDSLGLLELDQR	https://assays.cancer.gov/non-CPTAC-6078
non-CPTAC-6079	LVNEVTEFAK	https://assays.cancer.gov/non-CPTAC-6079
non-CPTAC-6080	AINPINTFTK	https://assays.cancer.gov/non-CPTAC-6080
non-CPTAC-6081	VLIEGSINSVR	https://assays.cancer.gov/non-CPTAC-6081
non-CPTAC-6082	AGSSEWLAVDGLVSPSNNSK	https://assays.cancer.gov/non-CPTAC-6082
non-CPTAC-6083	LFSGDVVLTAR	https://assays.cancer.gov/non-CPTAC-6083
non-CPTAC-6084	ELGIWEPLAVK	https://assays.cancer.gov/non-CPTAC-6084
non-CPTAC-6085	GTITVSAQELK	https://assays.cancer.gov/non-CPTAC-6085
non-CPTAC-6086	EHIEIIAPSPQR	https://assays.cancer.gov/non-CPTAC-6086
non-CPTAC-6088	YLTTAVITNK	https://assays.cancer.gov/non-CPTAC-6088
non-CPTAC-6089	DQIYDIFQK	https://assays.cancer.gov/non-CPTAC-6089

non-CPTAC-6090	IVIGYQSHADTATK	https://assays.cancer.gov/non-CPTAC-6090
non-CPTAC-6091	VLTLSDDLER	https://assays.cancer.gov/non-CPTAC-6091
non-CPTAC-6092	HGLLVPNNTTDQELQHIR	https://assays.cancer.gov/non-CPTAC-6092
non-CPTAC-6093	LSVISVEDPPQR	https://assays.cancer.gov/non-CPTAC-6093
non-CPTAC-6094	LEQDEYALR	https://assays.cancer.gov/non-CPTAC-6094
non-CPTAC-6095	GDFIALDLGGSSFR	https://assays.cancer.gov/non-CPTAC-6095
non-CPTAC-6096	ISRPGDSDDSR	https://assays.cancer.gov/non-CPTAC-6096
non-CPTAC-6097	VVNVSSIMSVR	https://assays.cancer.gov/non-CPTAC-6097
non-CPTAC-6098	ELEEIVQPIISK	https://assays.cancer.gov/non-CPTAC-6098
non-CPTAC-6099	VSLDVNHFAPDELTVK	https://assays.cancer.gov/non-CPTAC-6099
non-CPTAC-6100	GLPAPIEK	https://assays.cancer.gov/non-CPTAC-6100
non-CPTAC-6101	LLIYWASTR	https://assays.cancer.gov/non-CPTAC-6101
non-CPTAC-6102	ILPTLEAVAALGNK	https://assays.cancer.gov/non-CPTAC-6102
non-CPTAC-6103	IFVNDDR	https://assays.cancer.gov/non-CPTAC-6103
non-CPTAC-6104	LVVVGAVGVGK	https://assays.cancer.gov/non-CPTAC-6104
non-CPTAC-6105	IALDFQR	https://assays.cancer.gov/non-CPTAC-6105
non-CPTAC-6106	VYPLINR	https://assays.cancer.gov/non-CPTAC-6106
non-CPTAC-6107	ELIFEETAR	https://assays.cancer.gov/non-CPTAC-6107
non-CPTAC-6110	IFGVTTLDIVR	https://assays.cancer.gov/non-CPTAC-6110
non-CPTAC-6111	LFDAPEAPLPSR	https://assays.cancer.gov/non-CPTAC-6111
non-CPTAC-6113	IITLTGPTNAIFK	https://assays.cancer.gov/non-CPTAC-6113
non-CPTAC-6114	GSTAPVGGGAFPTIVER	https://assays.cancer.gov/non-CPTAC-6114
non-CPTAC-6115	FDAGELITQR	https://assays.cancer.gov/non-CPTAC-6115
non-CPTAC-6116	IVQAEGEAEAAK	https://assays.cancer.gov/non-CPTAC-6116
non-CPTAC-6117	VYTVDLGR	https://assays.cancer.gov/non-CPTAC-6117
non-CPTAC-6118	AAEIASSDSANVSSR	https://assays.cancer.gov/non-CPTAC-6118
non-CPTAC-6119	EAGLDLR	https://assays.cancer.gov/non-CPTAC-6119
non-CPTAC-6120	TGAAPIIDVVR	https://assays.cancer.gov/non-CPTAC-6120
non-CPTAC-6121	FIDTTSK	https://assays.cancer.gov/non-CPTAC-6121
non-CPTAC-6122	GTGIVSAPVPK	https://assays.cancer.gov/non-CPTAC-6122
non-CPTAC-6123	VVDPFSK	https://assays.cancer.gov/non-CPTAC-6123
non-CPTAC-6124	IGVLDEGK	https://assays.cancer.gov/non-CPTAC-6124
non-CPTAC-6126	VNIVPVIAK	https://assays.cancer.gov/non-CPTAC-6126
non-CPTAC-6127	ILVTGGSGLVGK	https://assays.cancer.gov/non-CPTAC-6127
non-CPTAC-6128	TIGTGLVTNTLAMTEEEK	https://assays.cancer.gov/non-CPTAC-6128
non-CPTAC-6129	LTIGSNLSIR	https://assays.cancer.gov/non-CPTAC-6129

Protein extraction & total protein quantitation. Solutions were prepared using LC-MS grade water and solvents, and analytical grade reagents. Cell line samples were lysed, extracted, denatured, and reduced using high-temperature incubation in sample extraction buffer (2% w:v sodium deoxycholate in 50 mM Tris-HCl, pH 8.1, 10 mM TCEP, 20 minutes, 99°C), followed by sonication (20% amplitude; 30 sec; pulse, 1s/1s) and an additional incubation (2 hours, 80°C). The concentration of total protein in each sample was then quantified using a Reducing Agent Compatible Pierce bicinchoninic acid (RAC-BCA) protein assay kit (Thermo Scientific, Cat # 23250) with a ThermoFisher MultiSkan Go spectrophotometer.

Tryptic digestion of cell line samples for multiplexed MRM-MS. Aliquots of 80 µg of denatured, reduced total protein were diluted to 0.4 µg/µL total protein for in-solution digestion in 25 mM ammonium bicarbonate (AmBic, pH 8). Each sample was then alkylated with 25 µL 90 mM iodoacetamide (IAA, in 25 mM AmBic) for 30 minutes while protected from light. Any remaining IAA was quenched with 20 µL 100 mM dithiothreitol (DTT, in 25 mM AmBic). Samples were diluted with 175 µL of 25 mM AmBic prior to digestion to ensure deoxycholate was not present at >1% w:v. Digestion was performed with trypsin (Worthington, TPCK Treated, 95% purity) dissolved in H2O, added at a substrate-to-enzyme ratio of 20:1, and allowed to incubate for 17±1 hours at 37°C. The digest was

then chilled on ice, spiked with an equimolar mixture of 54 SIS peptides (400 fmol on-column), and quenched with acidification using formic acid at a final concentration of 1%.

Solid Phase Extraction. Samples were centrifuged to pellet precipitated deoxycholate. The supernatant was collected for Solid Phase Extraction (Oasis SPE HLB 1cc cartridges, 10 mg sorbent), which was performed according to manufacturer directions on a vacuum manifold, as follows: (i) priming with 2 x 600 µL methanol rinse, (ii) equilibration with 2 x 600 µL H2O, 0.1% FA, (iii) sample loading in 600 µL H2O, 0.1% FA, (iv) washing with 3 x 600 µL H2O, 0.1% FA, (v) elution with 400 µL 55% ACN, 0.1% FA. Eluates were then dried under vacuum (LabConco CentriVap, 4°C) and reconstituted in 40 µL H2O, 0.1% FA.

Calibration curve & quality controls. The calibration curve was prepared by spiking an equimolar mix of unlabeled standard peptides in H2O, 1% FA at known quantities (0, 0.41, 1.02, 2.56, 6.40, 16.0, 40.0, 100, 250, 1000 fmol on-column) into previously digested BSA (0.01 ug on-column) spiked with the SIS peptide mixture (400 fmol on-column). A previously-quantified pool cell lysate sample was digested and re-quantified in parallel with each batch as a quality control.

Liquid chromatography. Samples and calibration standards were held at 4ºC in an autosampler until they were analyzed via 10 µL injections (20 µg total protein digest on-column) on an Agilent 1290 Infinity liquid chromatography system fitted with a Zorbax Eclipse plus C18 column (RRHD, 2.1x15mm, 1.8um) at 50ºC with a flow rate of 0.4mL/min. Elution was performed over a 48-minute method including a 46-minute gradient consisting of 2% to 7% B at 2 min, to 27% B at 44 min, to 45% B at 45 min, to 80% B at 45.5 min, followed by a wash at 80% B for 2 min, and then returning to 2% B at 48 min for a 2-minute equilibration (mobile phase A: H2O, 0.1% FA, mobile phase B: ACN, 0.1% FA).

Agilent 6495-QQQ-MS data acquisition MRM-MS analyses were performed on an in-line Agilent 6495B triple quadrupole mass spectrometer (Agilent Technologies, Santa Clara, CA). Mass spectra were acquired in positive ion mode (ESI capillary ion spray voltage, 3500 V; source gas temperature, 150°C; sheath gas temperature, 250°C; sheath gas flow, 11 L/min). The scheduled MRM method used a cycle time of 1100 ms and a 240 sec detection window. For each peptide, 5 MRM-MS transitions were monitored with a minimum of 1 peptide per protein and 1 associated SIS peptide (Sobsey et al, in preparation).

Data analysis. Data from the LC-MRM-MS assays were processed in Skyline-daily software (http://skyline.ms, Ver 4.1, MacCoss Lab, University of Washington, USA) (15), which was used for peak integration, linear range determination, and quantitation against the calibration curves to yield reproducible concentration data.

Statistical analysis. Multivariate statistical analysis was performed using the MetaboAnalyst webserver (www.metaboanalyst.ca) **(10)**. Values below the LLOQ were imputed as 1/5 of the minimum value. "Auto" data scaling, which expresses each variable measurement as the standard deviation from the variable's mean, was used to ensure equal weighting of features. MetaboAnalyst generated volcano plots, Principle Component Analysis, Partial Least Squares Discriminant Analysis, Variable Importance in the Projection (VIP) scores, heatmaps, and hierarchical clustering based on the normalized data.

References

1. Banerji U, Dean EJ, Pérez-Fidalgo JA, Batist G, Bedard PL, You B, *et al.* A Phase I Open-Label Study to Identify a Dosing Regimen of the Pan-AKT Inhibitor AZD5363 for Evaluation in Solid Tumors and in PIK3CA-Mutated Breast and Gynecologic Cancers. *Clin Cancer Res* 2018;**24**(9):2050-9 doi 10.1158/1078-0432.Ccr-17-2260.
2. Tate JG, Bamford S, Jubb HC, Sondka Z, Beare DM, Bindal N, *et al.* COSMIC: the catalogue of somatic mutations in cancer. *Nucleic acids research* 2019;**47**(D1):D941-D7.

3. Schmitt K, Däubener W, Bitter-Suermann D, Hadding U. A safe and efficient method for elimination of cell culture mycoplasmas using ciprofloxacin. *Journal of Immunological Methods* 1988;**109**(1):17-25 doi https://doi.org/10.1016/0022-1759(88)90437-1.
4. Popp R, Li H, LeBlanc A, Mohammed Y, Aguilar-Mahecha A, Chambers AG, *et al.* Immuno-Matrix-Assisted Laser Desorption/Ionization Assays for Quantifying AKT1 and AKT2 in Breast and Colorectal Cancer Cell Lines and Tumors. *Analytical Chemistry* 2017;**89**(19):10592-600 doi 10.1021/acs.analchem.7b02934.
5. Froehlich BC, Popp R, Sobsey CA, Ibrahim S, LeBlanc AM, Mohammed Y, *et al.* Systematic Optimization of the iMALDI Workflow for the Robust and Straightforward Quantification of Signaling Proteins in Cancer Cells. *Proteomics Clin Appl* 2020;**14**(5):e2000034 doi 10.1002/prca.202000034.
6. Sobsey CA, Froehlich B, Batist G, Borchers CH. Immuno-MALDI-MS for Accurate Quantitation of Targeted Peptides from Volume-Restricted Samples. Neuronal Cell Death: Springer; 2022. p. 203-25.
7. Domanski D, Murphy LC, Borchers CH. Assay development for the determination of phosphorylation stoichiometry using multiple reaction monitoring methods with and without phosphatase treatment: application to breast cancer signaling pathways. *Anal Chem* 2010;**82**(13):5610-20 doi 10.1021/ac1005553.
8. Froehlich BC, Gill HK, Joshi A, Goodlett DR. MS visualization and interpretation software (MS-VIS), a tool for visualizing mass spectra and analysing mass lists. *Rapid Communications in Mass Spectrometry* 2022;**36**(8):e9253 doi https://doi.org/10.1002/rcm.9253.
9. Spitzer M, Wildenhain J, Rappsilber J, Tyers M. BoxPlotR: a web tool for generation of box plots. *Nat Methods* 2014;**11**(2):121-2 doi 10.1038/nmeth.2811.
10. Pang Z, Chong J, Zhou G, de Lima Morais DA, Chang L, Barrette M, *et al.* MetaboAnalyst 5.0: narrowing the gap between raw spectra and functional insights. *Nucleic acids research* 2021;**49**(W1):W388-W96.
11. Szklarczyk D, Gable AL, Nastou KC, Lyon D, Kirsch R, Pyysalo S, *et al.* The STRING database in 2021: customizable protein–protein networks, and functional characterization of user-uploaded gene/measurement sets. *Nucleic acids research* 2021;**49**(D1):D605-D12.
12. Doncheva NT, Morris JH, Gorodkin J, Jensen LJ. Cytoscape StringApp: Network Analysis and Visualization of Proteomics Data. *J Proteome Res* 2019;**18**(2):623-32 doi 10.1021/acs.jproteome.8b00702.
13. Whiteaker JR, Halusa GN, Hoofnagle AN, Sharma V, MacLean B, Yan P, *et al.* CPTAC Assay Portal: a repository of targeted proteomic assays. *Nature methods* 2014;**11**(7):703-4.
14. Carr SA, Abbatiello SE, Ackermann BL, Borchers C, Domon B, Deutsch EW, *et al.* Targeted peptide measurements in biology and medicine: best practices for mass spectrometry-based assay development using a fit-for-purpose approach. *Mol Cell Proteomics* 2014;**13**(3):907-17 doi 10.1074/mcp.M113.036095.
15. Pino LK, Searle BC, Bollinger JG, Nunn B, MacLean B, MacCoss MJ. The Skyline ecosystem: Informatics for quantitative mass spectrometry proteomics. *Mass spectrometry reviews* 2020;**39**(3):229-44.
