# Supplementary material for: mTORC1-Driven Protein Translation Correlates with Clinical Benefit of Capivasertib within a Genetically Preselected Cohort of PIK3CA-Altered Tumors
Source: Cancer Res Commun. 2024 Aug 13;4(8):2058–74. doi: 10.1158/2767-9764.CRC-24-0113 (PMC11320025; doi:10.1158/2767-9764.CRC-24-0113)
Supplement: Supplementary Validation Data S1 — Supplementary Validation Data demonstrating the performance of the iMALDI-MS assay [file crc-24-0113_supplementary_validation_data_s1_suppsd1.pdf]

Supplemental Validation Data S1 - iMALDI-MS Validation Data

AKT iMALDI assay performance & optimization. The iMALDI assay for quantitation of AKT1 and AKT2 was implemented at the Segal Cancer Proteomics Centre and optimized, as previously described (6). Signal suppression was initially observed in samples treated with 2U/ug of phosphatase. However, it was shown that 1U/ug of phosphatase did not cause signal suppression and was sufficient to fully dephosphorylate 10 fmol of each of synthetic phosho-AKT1 peptide and phospho-AKT2 peptide in quality control samples with 10 µg of digested E. coli lysate. The antibody-bead conjugation protocol was modified from the published protocol to extend the incubation period to 12 hours at 4ºC, which improved signal-to-noise by 1.3x (t-test, $p<0.01$). Phosphorylation quality controls consisting of different levels of synthetic AKT1 peptide:phospho-AKT1 peptides (1:3, 1:1, 3:1) generated AKT1 and pAKT1 values within +/-20% of the known concentration. Typical calibration curves are presented in Figure S2.1. Following optimization, the lower limit of quantitation (LLOQ) for AKT1 and AKT2 were found to be <0.65 fmol on-spot (28 pg/10 µg total protein), which is equal to published values (4). Intra-run coefficients of variation (CVs) between replicates at the LLOQ were <20%. The linearity of the assay was consistently high ($R^2>0.95$).

AKT iMALDI assay quality controls. The quantitative performance of the standards and assay was validated through the re-analysis of known samples. FFPE samples of mouse xenograft of colorectal cancer 580 (Jewish General Hospital, Montreal, QC, Canada) that had been previously measured at the University of Victoria during assay development were remeasured. The values obtained at McGill were similar to those previously obtained during assay validation at the University of Victoria for both AKT1 (sample 1: 5.1 fmol vs. 5.9 fmol, sample 2: 6.4 fmol vs. 6.2 fmol) and AKT2 (sample 1: 0.8 fmol vs. 1.2 fmol, sample 2: 1.1 fmol vs. 1.6 fmol). Greater variability was observed in AKT2 concentrations due to the lower signal-to-noise ratio for this peptide.

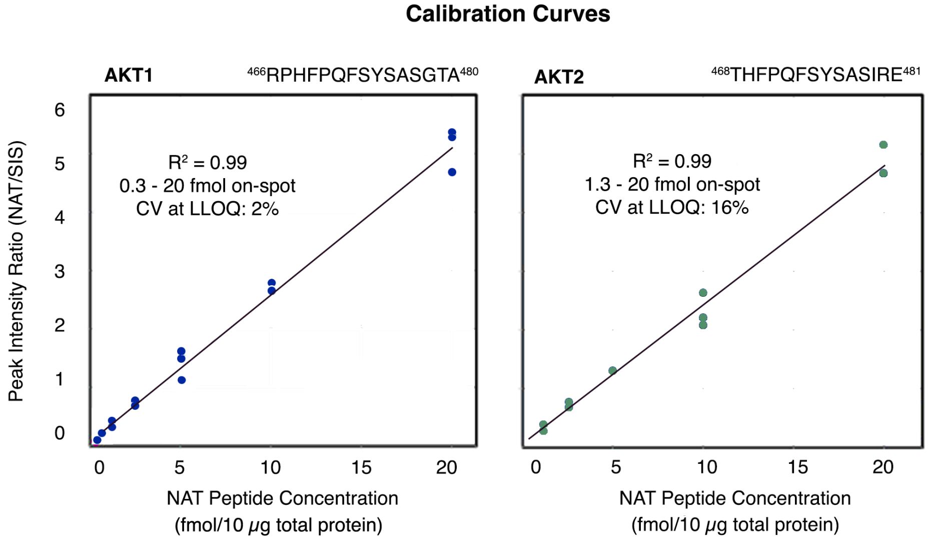

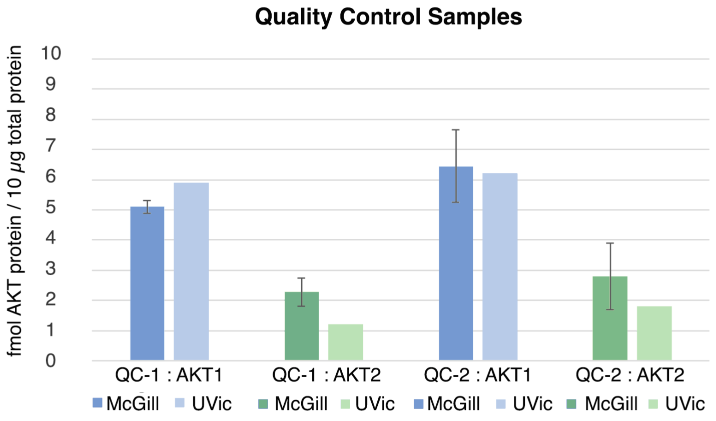

iMALDI Validation Data

(A) Sample calibration curves demonstrate the linear range of the assay based on the ratio of signal intensity between known quantities of unlabeled synthetic peptide (calibrants) versus a fixed quantity of SIS peptide (internal standard) immuno-enriched from E. coli lysate digest. Three (3) samples were analyzed per concentration level, plotted individually with a weighting of 1/x2. (B) Analysis of quality control samples.
