# Supplementary material for: mTORC1-Driven Protein Translation Correlates with Clinical Benefit of Capivasertib within a Genetically Preselected Cohort of PIK3CA-Altered Tumors
Source: Cancer Res Commun. 2024 Aug 13;4(8):2058–74. doi: 10.1158/2767-9764.CRC-24-0113 (PMC11320025; doi:10.1158/2767-9764.CRC-24-0113)
Supplement: Supplementary Validation Data S2 — Quality Assessment of the Global Proteomics Data [file crc-24-0113_supplementary_validation_data_s2_suppsd2.pdf]

Supplemental Validation Data S2 - Nano-LC-Orbitrap-MS Global Proteomics Data Quality Assessment

We performed specific statistical analyses to assess the quality of our complete global proteomics workflow and data processing. First, to assess the reproducibility of the experimental workflow, we compared data from 5 tumour samples for which we analyzed multiple replicates. The replicates consisted of 2 slides per tumour, extracted and analysed separately, but in the same analytical batch. For each slide, there were 2 technical replicates corresponding to supernatant samples from the AKT1 and AKT2 immunoenrichment steps. As shown in Figure S.3.1, related samples for tumours #2-5 cluster closely together, indicating that the data is highly reproducible, irrespective of the tumour slice used or the antibody used for enrichment. There is a greater difference between the slides from tumour #1; this may point to a difference in the original tumour material captured on the slide or a difference in what could be extracted from each slide. Since normalization, scaling, and batch integration steps were performed on the label-free quantitation data as described in the methods section, we also assessed the global proteome data for the possibility of batch effects. As shown in the Figure, no significant difference between batches was observed in PCA following data normalization.

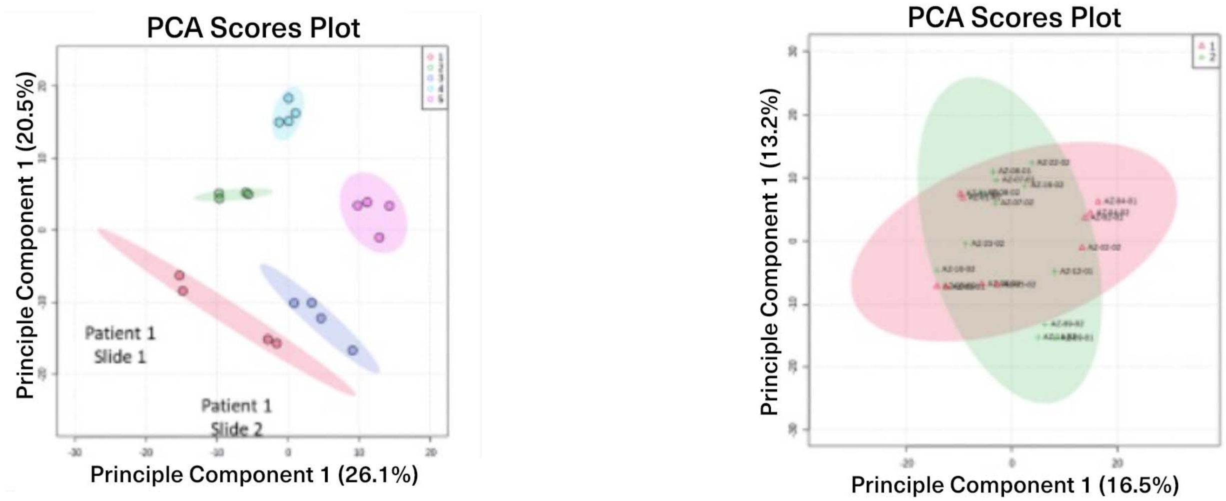

Quality control of processing steps of global proteome data

(Left) using PCA to assess clustering of related replicates (left, each dot represents a separate replicate and each color signifies a separate patient) and (Right) clustering of samples based on batch assignment (right, Batch 1 in red, Batch 2 in green). Figures generated with MetaboAnalyst webserver.
