# Supplementary material for: mTORC1-Driven Protein Translation Correlates with Clinical Benefit of Capivasertib within a Genetically Preselected Cohort of PIK3CA-Altered Tumors
Source: Cancer Res Commun. 2024 Aug 13;4(8):2058–74. doi: 10.1158/2767-9764.CRC-24-0113 (PMC11320025; doi:10.1158/2767-9764.CRC-24-0113)
Supplement: Supplementary Figure S1 — Individual protein concentrations measured by iMALDI-MS for each sample, comparison of measured PTEN concentration to IHC H-score [file crc-24-0113_supplementary_figure_s1_suppsf1.pdf]

Supplemental Figure S1 - Individual protein concentrations for patient samples

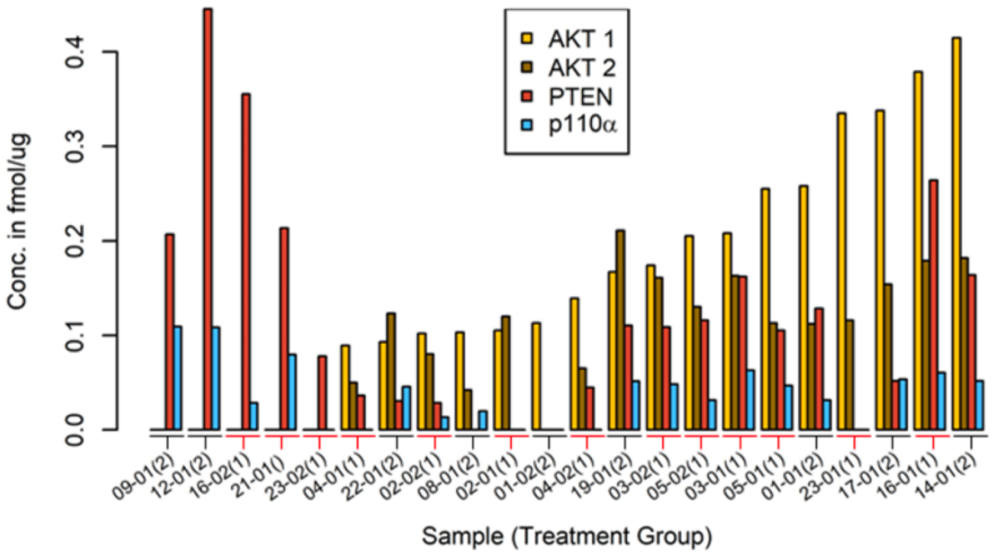

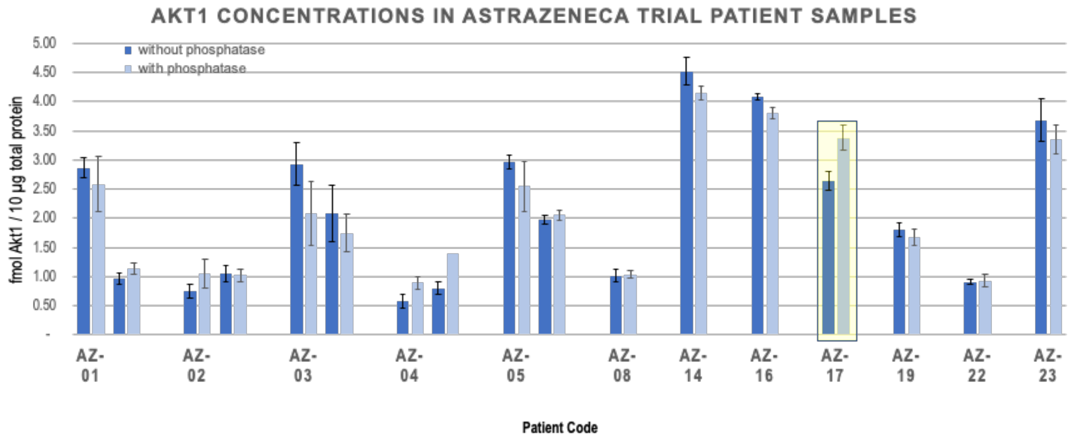

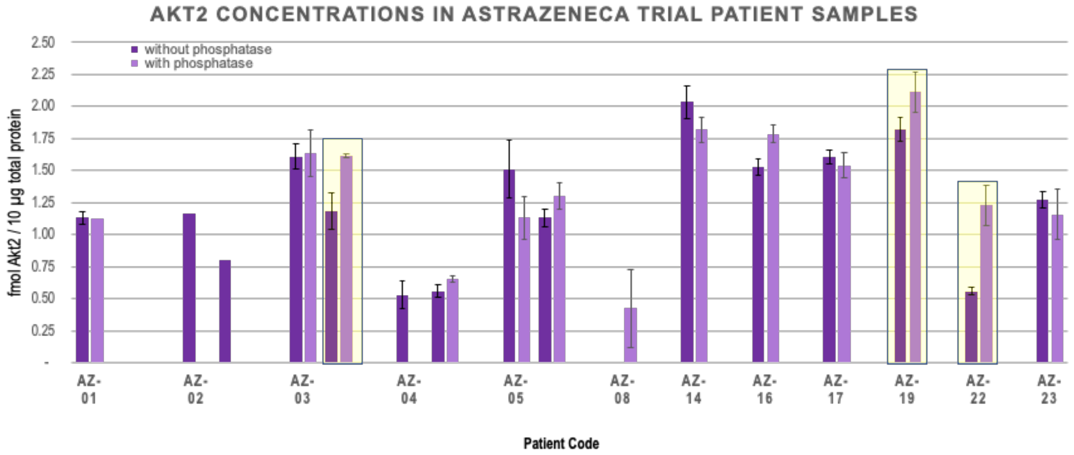

Figure S1.

(A) total AKT1, total AKT2, PTEN and PIK3CA p110a concentrations measured in patient samples using iMALDI-MS assays.

(B) AKT1 and AKT2 concentrations measured in patient samples.

(C) Non-phosphorylated AKT is measured before and after phosphatase treatment. Observed phosphorylation >30% is identified with a yellow highlight.
