# Supplementary material for: mTORC1-Driven Protein Translation Correlates with Clinical Benefit of Capivasertib within a Genetically Preselected Cohort of PIK3CA-Altered Tumors
Source: Cancer Res Commun. 2024 Aug 13;4(8):2058–74. doi: 10.1158/2767-9764.CRC-24-0113 (PMC11320025; doi:10.1158/2767-9764.CRC-24-0113)
Supplement: Supplementary Figure S2 — Individual protein concentrations measured by iMALDI-MS for PTEN vs. IHC H-score [file crc-24-0113_supplementary_figure_s2_suppsf2.pdf]

**Supplementary Figure S2. Comparison of PTEN concentration measured by iMALDI versus H-score**

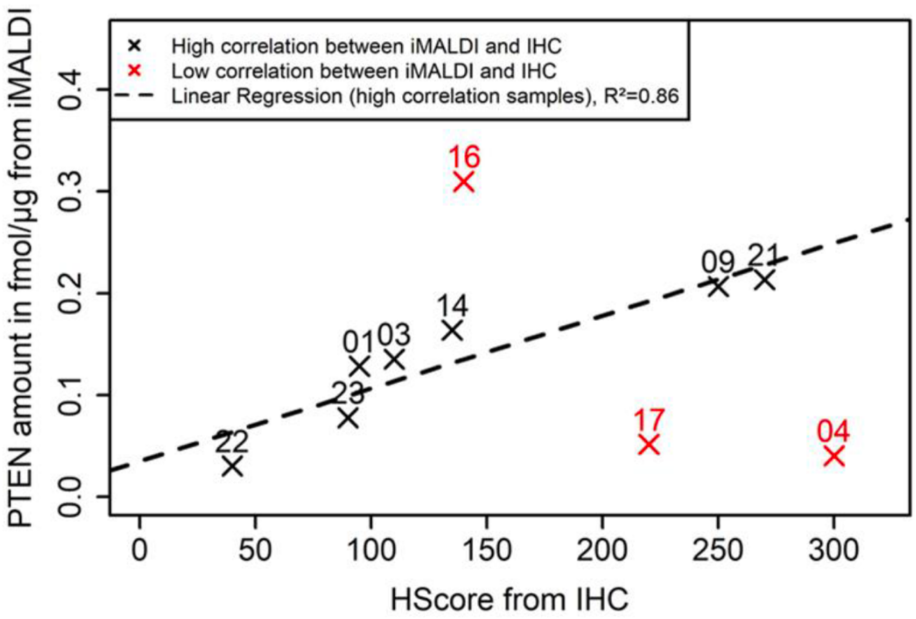


*Figure S2. PTEN concentration measured by iMALDI-MS assay in patient samples vs. IHC H-score for the same tumour. Where applicable, the average of two slices, analyzed as separate replicates is plotted. No correction is made to the iMALDI-measured protein concentration to account for tumour cellularity.*
