# Supplementary material for: mTORC1-Driven Protein Translation Correlates with Clinical Benefit of Capivasertib within a Genetically Preselected Cohort of PIK3CA-Altered Tumors
Source: Cancer Res Commun. 2024 Aug 13;4(8):2058–74. doi: 10.1158/2767-9764.CRC-24-0113 (PMC11320025; doi:10.1158/2767-9764.CRC-24-0113)
Supplement: Supplementary Figure S3 — Comparison of iMALDI and Global Proteomics results for primary vs metastatic samples [file crc-24-0113_supplementary_figure_s3_suppsf3.pdf]

Supplementary Figure S3.

Analysis of proteomic differences between samples from metastatic versus primary sites

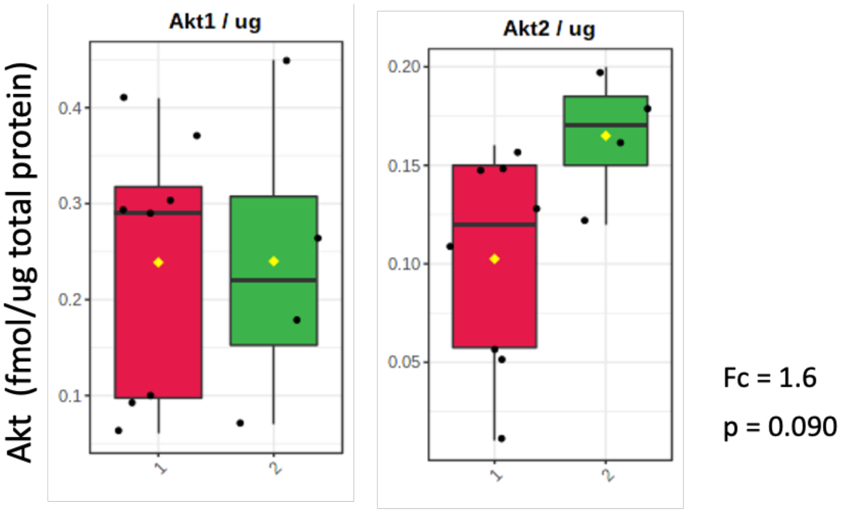

Figure S3.1. AKT concentration compared in tumour samples of primary (red) vs. metastatic (green) origin.

No difference in total AKT1 concentration is observed between the groups, though there is a non-significant trend toward increased AKT2 in the tumours of metastatic origin over primaries.

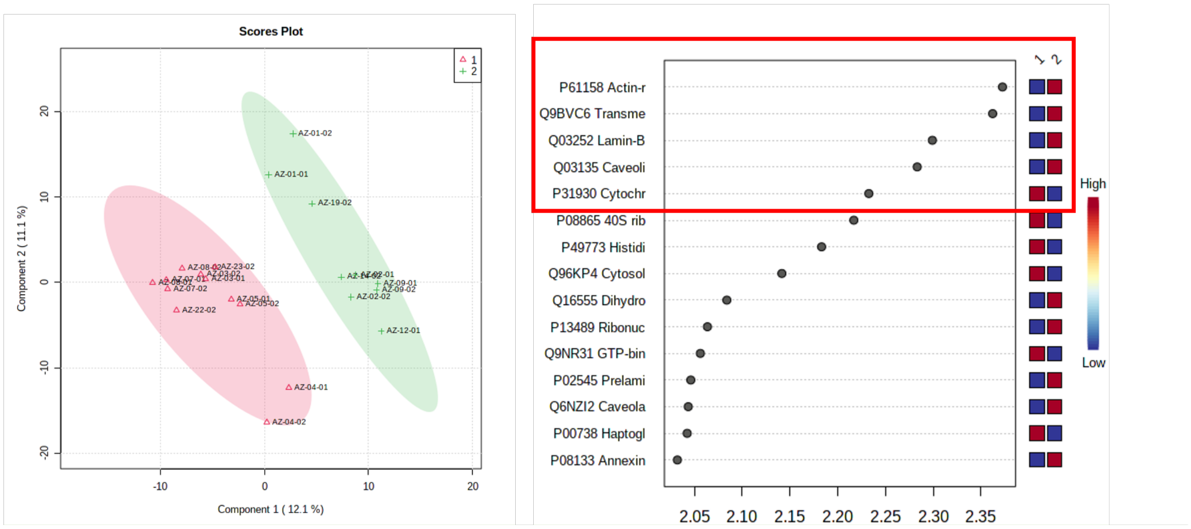

Figure S3.2. When separated by PLSDA (left), the proteins that best differentiate between patient tumours of primary (red) vs. metastatic (green) origin based on VIP score (right).

The identified proteins are consistent with the known biology of metastasis, and do not significantly overlap the proteins of interest in the current study.
