# Supplementary material for: mTORC1-Driven Protein Translation Correlates with Clinical Benefit of Capivasertib within a Genetically Preselected Cohort of PIK3CA-Altered Tumors
Source: Cancer Res Commun. 2024 Aug 13;4(8):2058–74. doi: 10.1158/2767-9764.CRC-24-0113 (PMC11320025; doi:10.1158/2767-9764.CRC-24-0113)
Supplement: Supplementary Figure S4 — Combination treatment of cell lines with capivasertib and an eIF2α inhibitor [file crc-24-0113_supplementary_figure_s4_suppsf4.pdf]

Supplementary Figure S4. Combination treatment of cell lines with capivasertib and an eIF2α inhibitor

Capivasertib (MedChemExpress, South Brunswick, USA) and ISRIB (Selleck Chemicals LLC, Houston, US) were dissolved in DMSO at concentrations of 30 mM and 10 mM respectively. Drugs were stored at -20ºC for a maximum of 3 months. ISRIB (trans-isomer, SelleckChem, S7400) is a selective eIF2α inhibitor and does not have global effects on translation, transcription, or mRNA stability in non-stressed cells (16).

As shown in Figure S10.1, a concentration matrix was used to testing combination treatment for synergistic effects. The matrix was replicated on each of 5 plates. After 72 hours of incubation with treatment, medium was replaced with 100 uL alamarBlue™ (Invitrogen) and incubated for ~30 minutes, as described above. An EnSpire® Multimode Plate Reader (PerkinElmer) was used to measure fluorescence with an excitation wavelength at 530–560 nm and an emission wavelength at 590 nm in the interior 60 wells of the plate. Multi-drug data was analyzed using SynergyFinder to compute synergy scores (17).

This approach was first applied to HCC-1428 using 5 independent replicates, measured by a standard Alamar blue cytotoxicity assay at 72 hours.

	1	2	3	4	5	6	7	8	9	10	11	12
A												
B	AKTi (nM)	9000	9000	9000	9000	9000	9000	9000	9000	9000	9000	
	EIF2i (nM)	9000	3000	1000	333	111	37	12	4.1	1.4	0	
C	AKTi (nM)	1500	1500	1500	1500	1500	1500	1500	1500	1500	1500	
	EIF2i (nM)	9000	3000	1000	333	111	37	12	4.1	1.4	0	
D	AKTi (nM)	250	250	250	250	250	250	250	250	250	250	
	EIF2i (nM)	9000	3000	1000	333	111	37	12	4.1	1.4	0	
E	AKTi (nM)	42	42	42	42	42	42	42	42	42	42	
	EIF2i (nM)	9000	3000	1000	333	111	37	12	4.1	1.4	0	
F	AKTi (nM)	6.9	6.9	6.9	6.9	6.9	6.9	6.9	6.9	6.9	6.9	
	EIF2i (nM)	9000	3000	1000	333	111	37	12	4.1	1.4	0	
G	AKTi (nM)	0	0	0	0	0	0	0	0	0	0	
	EIF2i (nM)	9000	3000	1000	333	111	37	12	4.1	1.4	0	
H												

Figure S10.1. Concentration matrix of 96-well plates used in cytotoxicity assays to test for synergistic effects of combination treatments

Synfinder calculated the synergy score and provided visualization of the results, as shown in Figure S10.2. Across all replicates, Synfinder's average reported synergy score was -1.19 +/- 3.067, with a max reported value for any area of the plot at 12.1. These numbers represent the excess response as compared to simply an additive effect between the drugs (e.g., a score of 10 represents a response 10% greater than expected). Therefore, no synergy was detected for HCC-1428.

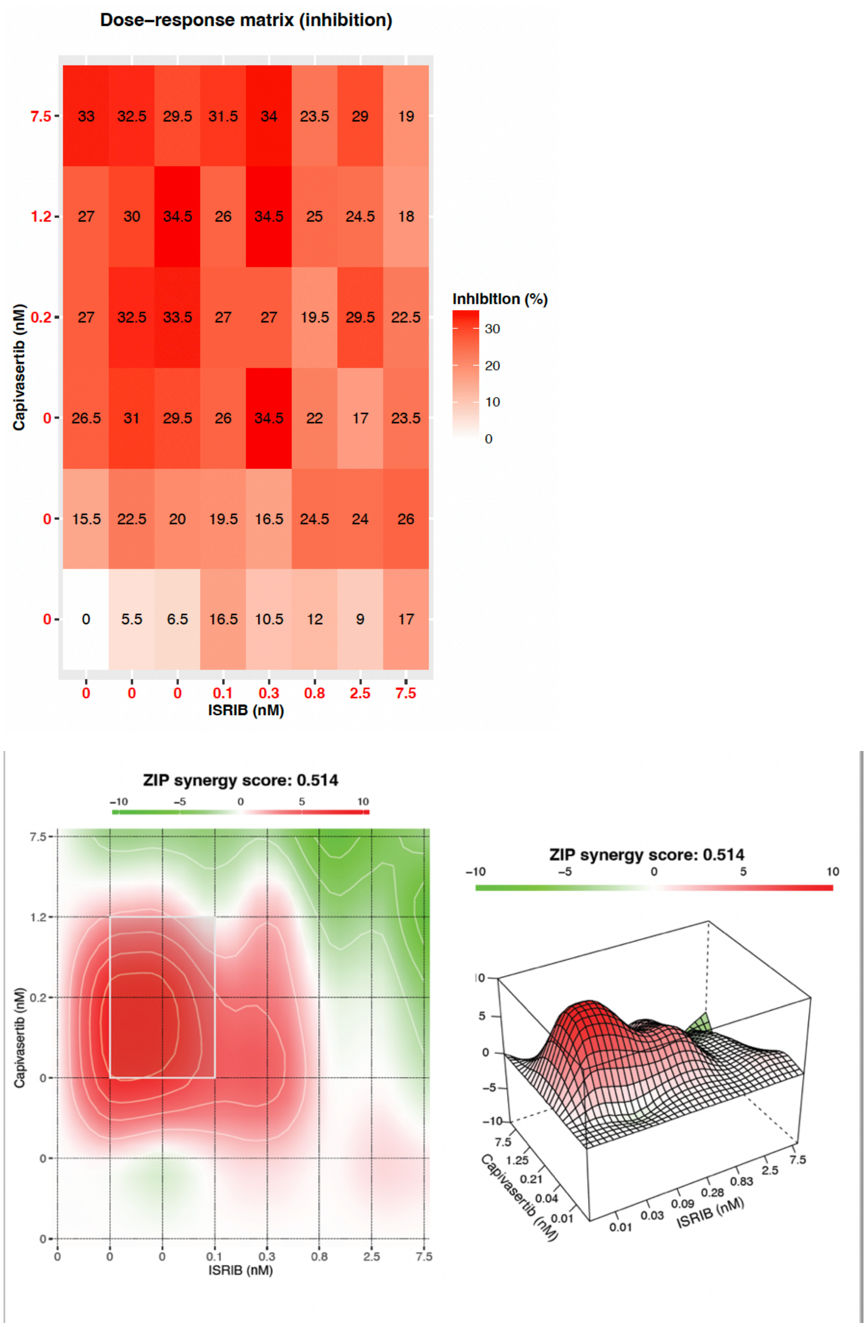

Figure S10.2. Synfinder scoring of synergy between capivasertib and ISRIB

References

16. Sidrauski C, Acosta-Alvear D, Khoutorsky A, Vedantham P, Hearn BR, Li H, *et al.* Pharmacological brake-release of mRNA translation enhances cognitive memory. *Elife* 2013;**2**:e00498 doi 10.7554/eLife.00498.

17. Ianevski A, Giri AK, Aittokallio T. SynergyFinder 3.0: an interactive analysis and consensus interpretation of multi-drug synergies across multiple samples. *Nucleic Acids Res* 2022;**50**(W1):W739-W43 doi 10.1093/nar/gkac382.
