# Supplementary material for: mTORC1-Driven Protein Translation Correlates with Clinical Benefit of Capivasertib within a Genetically Preselected Cohort of PIK3CA-Altered Tumors
Source: Cancer Res Commun. 2024 Aug 13;4(8):2058–74. doi: 10.1158/2767-9764.CRC-24-0113 (PMC11320025; doi:10.1158/2767-9764.CRC-24-0113)
Supplement: Supplementary Table S1 — Detailed results of the label-free quantitation [file crc-24-0113_supplementary_table_s1_suppst1.pdf]

Supplementary Table S1

Proteins expression differing between CB and NCB groups based on label-free quantitation data

Table S1. Proteins differing between CB vs. NCB groups (FC > 1.5, p<0.05)

PROTEIN ACCESSION	GENE NAME	FC (CB/NCB)	p value
P23526 Adenosylhomocysteinase	**AHCY**	0.534	0.00135
P02768 Serum albumin	**ALB**	1.976	0.02088
P07741 Adenine phosphoribosyltransferase	**APRT**	0.602	0.04590
Q07960 Rho GTPase-activating protein 1	**ARHGAP1**	0.609	0.02775
P59998 Actin-related protein 2/3 complex subunit 4	**ARPC4**	0.647	0.03223
P56134 ATP synthase subunit f, mitochondrial	**ATP5MF**	0.596	0.01905
P48047 ATP synthase subunit O, mitochondrial	**ATP5PO**	0.633	0.02377
P16152 Carbonyl reductase [NADPH] 1	**CBR1**	0.548	0.02675
P49368 T-complex protein 1 subunit gamma	**CCT3**	0.637	0.00527
P50990 T-complex protein 1 subunit theta	**CCT8**	0.476	0.00129
Q99829 Copine-1	**CPNE1**	0.538	0.02061
Q7Z4W1 L-xylulose reductase	**DCXR**	0.510	0.03662
Q16698 2,4-dienoyl-CoA reductase, mitochondrial	**DECR1**	0.586	0.02935
P38117 Electron transfer flavoprotein subunit beta	**ETFB**	0.474	0.00345
P55084 Trifunctional enzyme subunit beta, mitochondrial	**HADHB**	0.507	0.04801
P19367 Hexokinase-1	**HK1**	0.509	0.01396
P52597 Heterogeneous nuclear ribonucleoprotein F	**HNRNPF**	0.520	0.00457
P14866 Heterogeneous nuclear ribonucleoprotein L	**HNRNPL**	0.595	0.00296
P08238 Heat shock protein HSP 90-beta	**HSP90AB1**	0.590	0.00552
P04792 Heat shock protein beta-1	**HSPB1**	0.533	0.01080
P01859 Immunoglobulin heavy constant gamma 2	**IGHG2**	2.339	0.01448
P01834 Immunoglobulin kappa constant	**IGKC**	2.012	0.01815
P06312 Immunoglobulin kappa variable 4-1	**IGKV4-1**	2.945	0.00499
Q12905 Interleukin enhancer-binding factor 2	**ILF2**	0.591	0.01400
Q12906 Interleukin enhancer-binding factor 3	**ILF3**	0.606	0.00687
P17931 Galectin-3	**LGALS3**	0.597	0.04662
πP40926 Malate dehydrogenase, mitochondrial	**MDH2**	0.529	0.01071
Q15365 Poly(rC)-binding protein 1	**PCBP1**	0.629	0.02384
Q15366 Poly(rC)-binding protein 2	**PCBP2**	0.500	0.00345
Q15084 Protein disulfide-isomerase A6	**PDIA6**	0.655	0.01484
P35232 Prohibitin	**PHB1**	0.562	0.00503
Q99623 Prohibitin-2	**PHB2**	0.622	0.04948
P01833 Polymeric immunoglobulin receptor	**PIGR**	15.678	0.04660
P60900 Proteasome subunit alpha type-6	**PSMA6**	0.531	0.02568
P62906 60S ribosomal protein L10a	**RPL10A**	0.608	0.02355
P62913 60S ribosomal protein L11	**RPL11**	0.660	0.04438

P50914 60S ribosomal protein L14	**RPL14**	0.583	0.01523
Q07020 60S ribosomal protein L18	**RPL18**	0.607	0.02737
P46776 60S ribosomal protein L27a	**RPL27A**	0.508	0.02702
P39023 60S ribosomal protein L3	**RPL3**	0.594	0.02538
P05388 60S acidic ribosomal protein P0	**RPLP0**	0.635	0.00178
P62249 40S ribosomal protein S16	**RPS16**	0.534	0.01384
P08708 40S ribosomal protein S17	**RPS17**	0.657	0.00425
P62269 40S ribosomal protein S18	**RPS18**	0.654	0.02504
P15880 40S ribosomal protein S2	**RPS2**	0.503	0.00412
P61247 40S ribosomal protein S3a	**RPS3A**	0.657	0.00425
P61247 40S ribosomal protein S3a	**RPS3A**	0.656	0.00728
P46781 40S ribosomal protein S9	**RPS9**	0.602	0.01431
Q9NR31 GTP-binding protein SAR1a	**SAR1A**	0.563	0.01391
P31040 Succinate dehydrogenase [ubiquinone] flavoprotein subunit, mitochondrial	**SDHA**	0.532	0.02468
Q00325 Phosphate carrier protein, mitochondrial	**SLC25A3**	0.450	0.00156
Q13630 GDP-L-fucose synthase	**TSTA3**	0.618	0.02588
P49411 Elongation factor Tu, mitochondrial	**TUFM**	0.396	0.00069
P13010 X-ray repair cross-complementing protein 5	**XRCC5**	0.647	0.02883
