# Supplementary material for: mTORC1-Driven Protein Translation Correlates with Clinical Benefit of Capivasertib within a Genetically Preselected Cohort of PIK3CA-Altered Tumors
Source: Cancer Res Commun. 2024 Aug 13;4(8):2058–74. doi: 10.1158/2767-9764.CRC-24-0113 (PMC11320025; doi:10.1158/2767-9764.CRC-24-0113)
Supplement: Supplementary Table S2 — Proteins expression differences between capivasertib-resistant vs. capivasertib-sensitive cells quantified by validated MRM-MS assays as compared to the profile observed in patient samples [file crc-24-0113_supplementary_table_s2_suppst2.pdf]

Supplementary Table S2. Proteins quantified in breast cancer cell lines

Table S3. Proteins expression differences between capivasertib-resistant vs. capivasertib-sensitive cells as compared to the profile observed in patient samples

VIP Ranking	Protein	Fold-change (resistant vs. sensitive)	p-value	FDR-adjusted p-value	Expression in resistant vs. sensitive	Expression in NCB vs. CB
1	CBR1	0.16	0.0049	0.014	low	high
2	HSP90AB1	0.12	0.0200	0.047	low	high
3	PHB2	2.73	0.0001	0.002	high	high
4	MTOR	5.16	0.0008	0.005	high	pred. activated
5	HSPB1	0.27	0.0023	0.009	low	high
6	EIF4E	1.98	0.0001	0.002	high	pred. activated
7	SEPTIN2	2.93	0.0017	0.007	high	pred. activated
8	PHB1	2.14	0.0049	0.014	high	high
9	RPL27A	2.68	0.0002	0.002	high	high
10	RPS2	2.22	0.0002	0.002	high	high
11	RPL3	3.54	0.0031	0.011	high	high
12	RPS9	2.85	0.0007	0.005	high	high
13	RPS3A	2.20	0.0011	0.006	high	high
14	CPNE1	5.68	0.0008	0.005	high	high
15	CCT3	1.57	0.0018	0.007	high	high
16	EIF3E	2.82	0.0039	0.013	high	pred. activated
17	ARPC4	2.68	0.0132	0.035	high	high
18	HNRNPL	1.47	0.0199	>0.05	high	high
19	EIF2S1	1.51	0.1374	>0.05	high	pred. activated
20	LGALS3	0.36	0.0954	>0.05	low	high
21	XRCC5	2.41	0.0834	>0.05	high	high
22	EIF2AK3	2.71	0.0593	>0.05	high	pred. activated
23	PDIA6	1.44	0.0266	>0.05	high	high
24	HADHB	2.04	0.0373	>0.05	high	high
25	TSTA3	3.18	0.0252	>0.05	high	high
26	PIK3CB	1.68	0.0308	>0.05	high	-
27	PIGR	0.47	0.3024	>0.05	low	low
28	PCBP1	0.81	0.2971	>0.05	no change	high
29	TUFM	1.32	0.1445	>0.05	no change	high

VIP Ranking	Protein	Fold-change (resistant vs. sensitive)	p-value	FDR-adjusted p-value	Expression in resistant vs. sensitive	Expression in NCB vs. CB
30	ILF2	1.63	0.1303	>0.05	high	high
>30	ALB	0.08	0.4066	>0.05	low	low
>30	ARHGAP1	3.15	0.0652	>0.05	high	high
>30	ATF4	1.01	0.7743	>0.05	no change	pred. activated
>30	EIF4A1	1.53	0.2321	>0.05	high	pred. activated
>30	EIF5	2.02	0.1280	>0.05	high	pred. activated
>30	ETFB	1.06	0.8466	>0.05	no change	high
>30	HSPA5	0.87	0.6022	>0.05	no change	-
>30	ILF3	1.30	0.3118	>0.05	no change	high
>30	KRAS G12V	1.39	0.5860	>0.05	no change	-
>30	MAPK1	1.60	0.1352	>0.05	high	pred. activated
>30	MDH2	1.83	0.1188	>0.05	high	high
>30	S6K1	1.24	0.6434	>0.05	no change	pred. activated
