# Supplementary material for: mTORC1-Driven Protein Translation Correlates with Clinical Benefit of Capivasertib within a Genetically Preselected Cohort of PIK3CA-Altered Tumors
Source: Cancer Res Commun. 2024 Aug 13;4(8):2058–74. doi: 10.1158/2767-9764.CRC-24-0113 (PMC11320025; doi:10.1158/2767-9764.CRC-24-0113)
Supplement: Supplementary Table S3 — Detailed results of pathway mapping by Qiagen IPA [file crc-24-0113_supplementary_table_s3_suppst3.pdf]

Supplementary Table S3. Proteins mapping to reported QIAGEN pathways

Table S2.1. Proteins mapped to top 8 QIAGEN IPA-defined differentially-regulated pathways and the direction of their regulation

Ingenuity Canonical Pathways	Proteins in pathway	Upregulated (NCB / CB)	Downregulated (NCB / CB)	Molecules
LXR/RXR Activation	123	0	12 (10%)	AGT, AHSG, ALB, AMBP, APOA2, APOH, CLU, GC, KNG1, ORM1, TF, TTR
FXR/RXR Activation	126	0	12 (10%)	AGT, AHSG, ALB, AMBP, APOA2, APOH, CLU, GC, KNG1, ORM1, TF, TTR
EIF2 Signaling	224	14 (6%)	0	EIF4A1, RPL10A, RPL14, RPL18, RPLP0, RPS11, RPS16, RPS17, RPS18, RPS2, RPS3A, RPS4X, RPS7, RPS9
Regulation of eIF4 and p70S6K Signaling	179	10 (6%)	0	EIF4A1, RPS11, RPS16, RPS17, RPS18, RPS2, RPS3A, RPS4X, RPS7, RPS9
mTOR Signaling	212	10 (5%)	0	EIF4A1, RPS11, RPS16, RPS17, RPS18, RPS2, RPS3A, RPS4X, RPS7, RPS9
Acute Phase Response Signaling	185	0	9 (5%)	AGT, AHSG, ALB, AMBP, APOA2, APOH, ORM1, TF, TTR
Clathrin-mediated Endocytosis Signaling	193	0	6 (3%)	ALB, APOA2, CLTC, CLU, ORM1, TF
Coronavirus Pathogenesis Pathway	203	10 (5%)	2 (1%)	AGT, KNG1, NPM1, RPS11, RPS16, RPS17, RPS18, RPS2, RPS3A, RPS4X, RPS7, RPS9

Table S2.2. QIAGEN IPA-proposed upstream regulators, predicted activation state, and associated differential proteins

Upstream Regulator	Molecule Type	Predicted Activation State (NCB / CB)	p-value of overlap	Target Molecules in Dataset
MYCN	transcription regulator	activated	5.39E-15	CLU, EEF1G, EIF4A1, NME1, NPM1, PHB1, RPL18, RPLP0, RPS16, RPS17, RPS2, RPS3A, RPS4X, RPS7, RPS9, TUBB, TUFM
MYC	transcription regulator	activated	5.16E-14	AHCY, ALB, CCT3, CLU, EFEMP1, EIF4A1, HADHA, HADHB, HNRNPAB, HSPB1, NME1, NPM1, PHB1, PRDX3, RAB10, RPL10A, RPL14, RPL18, RPLP0, RPS11, RPS16, RPS17, RPS18, RPS2, RPS7, RPS9, TF
TCR	complex		1.45E-09	MDH2, NME1, PHB1, RPL10A, RPLP0, RPS16, RPS17, RPS2, RPS3A, RPS4X, SDHA, SLC25A3, TUFM
MAPT	other		1.46E-09	ALB, CCT8, CLTC, EEF1G, HK1, NME1, PEBP1, PRDX3, RAB10, RPLP0, RPS16, SPTAN1, TUBA4A, TUBB, TUFM
YAP1	transcription regulator		7.73E-09	RPL10A, RPL14, RPL18, RPLP0, RPS11, RPS16, RPS17, RPS18, RPS2, RPS7, RPS9, TUBB
RICTOR	other	inhibited	9.47E-09	ATP5MF, ATP5PO, RPL10A, RPL14, RPL18, RPLP0, RPS11, RPS18, RPS2, RPS9, SDHA
APP	other		8.76E-08	ALB, CD59, CLTC, CLU, EEF1G, HK1, HSPB1, KNG1, NME1, PEBP1, RAN, SPTAN1, TTR, TUBA4A, TUBB, TUFM, YWHAB
TP53	transcription regulator		1.35E-07	AHCY, ALB, ANXA2, CD59, CLTC, CLU, EIF4A1, GC, HADHA, HADHB, HSPB1, LASP1, MDH2, NME1, NPM1, ORM1, PRDX3, RAN, RPN1, RPS16, RPS18, SDHA, SERPINC1, TUBB
Lh	complex	activated	6.84E-07	PGRMC1, PHB1, RPL10A, RPS11, RPS16, RPS17, RPS18, RPS2, RPS7, RPS9
